# Chronotherapy with Cinacalcet has a striking effect on inhibition of parathyroid gland proliferation in rats with secondary hyperparathyroidism

**DOI:** 10.1371/journal.pone.0316675

**Published:** 2025-01-06

**Authors:** Søren Egstrand, Maria Lerche Mace, Marya Morevati, Lars Henning Engelholm, Jesper Skovhus Thomsen, Annemarie Brüel, Klaus Olgaard, Ewa Lewin

**Affiliations:** 1 Nephrological Department, Herlev Hospital, University of Copenhagen, Copenhagen, Denmark; 2 Nephrological Department, Rigshospitalet, University of Copenhagen, Copenhagen, Denmark; 3 Biotech Research and Innovation Centre, University of Copenhagen, Copenhagen, Denmark; 4 Finsen Laboratory, University of Copenhagen, Copenhagen, Denmark; 5 Department of Biomedicine, Aarhus University, Aarhus, Denmark; University of Vermont College of Medicine, UNITED STATES OF AMERICA

## Abstract

Secondary hyperparathyroidism (sHPT) is a significant clinical complication of CKD leading to bone abnormalities and cardiovascular disease. Current treatment based on activating the parathyroid calcium-sensing receptor (CaSR) using calcimimetics such as Cinacalcet, aims to decrease plasma PTH levels and inhibit the progression of parathyroid hyperplasia. In the present study, we found significant diurnal rhythmicity of *Casr*, encoding the Cinacalcet drug target in hyperplastic parathyroid glands (p = 0.006). In rats with sHPT, Cinacalcet treatment timed prior to the acrophase of *Casr* expression (chronotherapy: *Cina1*) was compared with the usual timing of treatment early in the active phase (conventional: *Cina2*). Without Cinacalcet treatment, induction of sHPT resulted in a significant increase in parathyroid proliferation in terms of Ki-67^+^ cells compared to that of control rats (p = 0.001). Conventional Cinacalcet treatment (*Cina2*) did not significantly reduce Ki-67 index compared to untreated rats with sHPT (p = 0.09). In contrast, chronotherapy treatment (*Cina1*) resulted in a marked inhibition of parathyroid proliferation by Ki-67^+^ cells compared to untreated rats with sHPT (p = 0.0001). We found significantly reduced parathyroid Ki-67 index using chronotherapy compared to conventional timing of Cinacalcet (*Cina1* vs. *Cina2*: 0.92±0.14% vs. 2.46±0.37%, p = 0.006). Transcriptomic analysis showed that the reduced proliferation of *Cina1* was associated with downregulation of genes involved in mitotic activity, together with an increased adaptive response of energy metabolism, as evident from upregulated pathways of *Oxidative phosphorylation* and *TCA cycle* compared to the untreated uremic group. Conclusively, it is shown that the inhibitory effect of Cinacalcet on parathyroid cell proliferation is markedly impacted by the timing of administration, suggesting a possible benefit of using chronotherapy in Cinacalcet treatment of sHPT.

## Introduction

Living on a planet with a 24 h day-night rhythm, evolutionary pressure has led to the development of molecular circadian clock mechanisms conserved from cyanobacteria to plants and animals. Ambient light is the main entrainment cue to the circadian clock. In mammals information on ambient light is transmitted from light sensitive cells in the retina to the highly specialized master pacemaker located in the hypothalamic suprachiasmatic nucleus. This master clock synchronizes the peripheral circadian clocks running in numerous tissues in which these endogenous clocks regulate an estimated 8–10% of all expressed genes specific to the given cell-type [1, 2]. In the field of chronobiology, time is measured in *Zeitgebertime* (ZT) denoting hours since light onset. There is a rapidly growing interest in the circadian rhythms in all aspects of human biology and an increasing acknowledgement that the timing of blood sampling and drug administration can have a huge impact on the reported results and pharmaceutical effects, respectively [3]. Chronotherapy is the principle of timing administration of drugs to the diurnal rhythms of the drug target to optimize the treatment effects and reduce side-effects and drug interactions [3].

We have recently shown that an internal circadian clock operates in the parathyroid glands [4]. We found that 6.85% of all genes expressed in the parathyroid glands of the mouse were significantly rhythmic. Furthermore, numerous parathyroid signature genes, including the calcium-sensing receptor gene, *Casr*, showed significant 24 h rhythmicity, which was abolished upon disturbance of the parathyroid circadian clock in a transgenic mouse model with knockdown of the core circadian clock activator *Bmal1* [5].

Proliferation is very limited in the parathyroid gland under normal conditions [6, 7], but increases dramatically in secondary hyperparathyroidism (sHPT) caused by chronic kidney disease (CKD). Clinically, sHPT is associated with low bone density and vascular calcification. sHPT in CKD occurs with a combination of functional and structural changes in the parathyroid glands resulting in increased parathyroid hormone (PTH) biosynthesis and secretion as well as in parathyroid hyperplasia [8].

Cinacalcet is a calcimimetic inducing positive allosteric modulation of the CaSR used once daily when treating advanced sHPT [9]. Cinacalcet has been shown to inhibit parathyroid proliferation in research animals [10] and the EVOLVE trial has shown a decrease in uncontrolled hyperparathyroidism with a reduced need for surgical parathyroidectomy upon Cinacalcet treatment [11]. Moreover, studies have shown that Cinacalcet treatment led to a decrease in parathyroid gland size as evaluated by ultrasonography [12–15]. Orally administrated Cinacalcet exhibits a short time to maximal plasma concentration followed by decline to baseline levels resulting in a relatively brief peak in plasma concentration and a corresponding decreased PTH secretion [16–18].

In the current investigation, it is shown that the gene expression of the target molecule of Cinacalcet, the parathyroid *Casr*, exhibited marked diurnal variation in rats with sHPT. The expression was increased in the inactive resting period and decreased in the active period. Therefore, we examined whether there was a beneficial effect of adapting the timing of Cinacalcet administration to match the peak expression of the *Casr*.

## Methods

### Study design: *Casr* gene expression over 24 h

A total of 44 male Wistar rats (Charles River, Germany) at 7-weeks of age were acclimatized for two weeks at our facility in a 12-hour light-dark cycle (lights on at 6.00 a.m. local time [= ZT0] and off at 6 p.m. [= ZT12]) and fed a standard diet (0.9% calcium, 0.5% phosphorus, and 1050 IU/kg vitamin D) with *ad libitum* access to water and food. After acclimatization, CKD was induced by one-step 5/6 nephrectomy [19] and rats were switched to a high phosphorus diet (0.9% calcium, 1.4% phosphorus, and 600 IU/kg vitamin D) for 8 weeks. At 4-hour intervals, 7–8 rats were anesthetized with Pentobarbital (Nycomed-DAK, Denmark), 50 mg/ml, at dose of 1 μl/g rat, and parathyroid glands were harvested by microdissection and immediately frozen in liquid nitrogen. Rats investigated in the dark period were carefully not exposed to blue light by using red light during anesthesia and covering the eyes once anesthetized. Rats were euthanized using a cardiac injection of Pentobarbital (Nycomed-DAK, Denmark), 200 mg/ml, at a dose of 1 ml.

### Study design: Cinacalcet chronotherapy

A total of 60 male Wistar rats (Charles River, Germany) at 7-weeks of age were acclimatized for two weeks at our facility in a 12-hour light-dark cycle and fed a standard diet (0.9% calcium, 0.5% phosphorus, and 1050 IU/kg vitamin D) with *ad libitum* access to water and food. After acclimatization, CKD was induced by one-step 5/6 nephrectomy [19] in 40 rats and the food were switched to a high phosphorus diet (0.9% calcium, 1.4% phosphorus, and 600 IU/kg vitamin D). After four weeks of uremia, 20 CKD rats were assigned to two treatment groups: *Cina1* and *Cina2* receiving Cinacalcet (STADA Nordic, Herlev, Denmark) at a dose of 2.5 mg once daily by oral gavage at either morning (ZT2, N = 10) or evening (ZT14, N = 10), respectively. This dose was based on previously published studies and a pilot study in rats showing a 30% decrease in PTH levels similar to a common endpoint in clinical studies [20]. The remaining 20 untreated CKD rats were assigned to morning (*PNX1*, N = 10) or evening (*PNX2*, N = 10) investigation. Likewise, the 20 normal control rats were assigned to morning (*ctrl1*, N = 10) or evening (*ctrl2*, N = 10) investigation. After three weeks of treatment, rats were investigated at the assigned time point, that is, morning (ZT2) for groups: *Cina1*, *PNX1*, and *ctrl1*, and evening (ZT14) for groups: *Cina2*, *PNX2*, and *ctrl2*. For treatment groups, investigation was performed 24 h after last treatment administration. Rats were anesthetized with Pentobarbital (Nycomed-DAK, Denmark), 50 mg/ml, at dose of 1 μl/g rat and blood was drawn from orbital venous plexus. Then the left parathyroid gland was harvested by microdissection and immediately frozen in liquid nitrogen while the right thyroid containing the right parathyroid gland was excised and fixed in 10% formalin. Aorta thoracalis was immediately frozen in liquid nitrogen. The left femoral bone was stripped for soft tissue and stored in 70% EtOH for μCT. Rats were euthanized using a cardiac injection of Pentobarbital (Nycomed-DAK, Denmark), 200 mg/ml, at a dose of 1 ml.

### Biochemistry

Blood urea nitrogen (BUN), creatinine, phosphate, and total calcium were analyzed using a Cobas 8000 biochemistry analyzer (Roche Diagnostics, Basel, Switzerland) on plasma samples stored at −20°C and thawed only once. Ionized Ca^2+^ was analyzed immediately after blood sampling on an ABL90 FLEX gas analyzer (Radiometer, Brønshøj, Denmark). PTH was measured by the rat bioactive PTH ELISA assay (Quidel, San Diego, USA). In our lab, the intra- and inter-assay variations are 4% and 9%, respectively [21]. FGF23 was measured by the intact FGF23 ELISA assay (Kainos Laboratories, Japan). In our lab, the intra-and inter-assay variations were 2.5% and 5%, respectively [22].

### Immunohistochemistry

Right thyroparathyroid specimens from were fixed in 10% formalin and embedded in paraffin. Formalin-fixed, paraffin-embedded thyroparathyroid tissue sections were cut in 3.5-μm-thick sections and mounted on aminopropyltriethoxysilane-coated glass slides. Sections were available from 9 *ctrl1* rats, 7 *ctrl2* rats, 7 *PNX1* rats, 7 *PNX2* rats, 10 *Cina1* rats, and 9 *Cina2* rats. Tissue sections were placed in a heating cabinet at 60°C for one hour, and then deparaffinized and rehydrated using Tissue-Tek Tissue Clear (Sakura, Alphen aan den Rijn, The Netherlands) and decreasing concentrations of ethanol, followed by 5 min rinsing in water. The sections were treated with 0.1 mol/L citrate, pH 6.0, in a microwave oven for 10 min for antigen retrieval, followed by blocking of endogenous peroxidase activity with 1% H_2_O_2_ for 15 min. Immunostainings were performed using rabbit anti-Ki67 monoclonal Ab (ab16667, Abcam, Cambridge, UK) diluted 1:100 in Background Reducing Antibody Diluent (Agilent, California, USA). Sections were incubated with primary antibodies overnight at 4°C, washed in TBS-T (50 mM Tris, 150 mM NaCl, 0.5% Triton X-100, pH 7.6), and incubated 45 min with EnVision + System HRP Labelled Polymer Anti-Rabbit IgG (Agilent, California, USA). Chromogen staining was performed using the NovaRED HRP substrate kit (VWR, Pennsylvania, USA). Nuclear counterstaining was done with Mayer’s hematoxylin (Histolab, Askim, Sweden). Then sections were dehydrated with increasing concentrations of ethanol and mounted using Tissue-Tek Tissue Mount (Sakura, Alphen aan den Rijn, The Netherlands). Slides were scanned on a Nanozoomer 2.0 HT Digital Pathology slide scanner (Hamamatsu, Shizuoka, Japan).

### Quantification

The scanned slides were analyzed using Qupath v.0.2.3 [23]. Region of analysis was delimited by the fibrous capsule of the parathyroid glands excluding adjacent thyroid tissue. Intraparathyroid blood vessels were excluded for analysis. For detection of positive DAB staining, the positive cell detection function was used at default settings except for “Sigma”, which was set at 0.8, “Minimal Nucleus Size”, which was set at 8 μm, and threshold, which was set at 0.4.

### Micro-Computed Tomography (μCT)

Left femoral bone was available from 10 *ctrl1* rats, 10 *ctrl2* rats, 8 *PNX1* rats, 7 *PNX2* rats, 10 *Cina1* rats, and 10 *Cina2* rats. The distal metaphysis and mid-diaphysis of the left femora were scanned in a desktop μCT scanner (Scanco μCT 35; Scanco Medical, Brüttiselen, Switzerland). Scans were conducted in high resolution mode (1000 projections/180°) with isotropic voxel size of either 3.5 μm (distal metaphysis) or 6.0 μm (mid-diaphysis), x-ray voltage of 55 kV, current of 145 μA, and an integration time of 800 ms. Beam hardening effects were reduced by using a 0.5 mm aluminum filter. Volumes of interest (VOIs) were drawn semi-automatically using the software provided with the scanner. The femoral metaphyseal VOI was set 200 μm above the distal part of the growth plate and extended 1000 μm further above, thus excluding primary spongiosa and including trabecular bone only. The femoral mid-diaphyseal VOI was set as a 1398-μm-high region centered on the mid-point of the bone shaft, thus containing cortical bone only. The three-dimensional dataset was low-passed-filtered using a Gaussian filter (σ = 0.8, support = 1) and segmented with a fixed threshold filter of 510 HA/cm^3^. The assessment of the bone microstructure using μCT was performed in accordance with current guidelines [24]. Quality assurance was performed by scans of the solid-state calibration phantom, which were provided with the scanner. The CV of distal femoral metaphyseal bone volume/tissue volume determined with the μCT scanner is 1.8% in our laboratory (the same sample measured 10 times).

### Aortic calcium quantification

Ascending aorta was available from 8 *ctrl1* rats, 10 *ctrl2* rats, 7 *PNX1* rats, 7 *PNX2* rats, 9 *Cina1* rats, and 10 *Cina2* rats. To assess the level of blood vessel calcification, calcium content of the ascending aorta was quantified and normalized to tissue dry weight (μg Calcium/mg aorta tissue). Aortic calcium content was determined by the o-cresolphthalein method [22]. Briefly, a section of the aorta was lyophilized for 24 hours to determine dry weight followed by decalcification in 1 M HCl for three days and the calcium content of the supernatant was determined using a commercial calcium colorimetric assay (MAK022-1KT, Sigma-Aldrich, Missouri, USA).

### Quantitative RT-PCR

Total RNA was extracted using TRIzol (Sigma-Aldrich, St Louis, MO, USA) according to the manufacturer’s instructions. First-strand cDNA was synthesized from 1.0 μg of RNA with Superscript III cDNA kit (Invitrogen, MA, USA). Jumpstart (Sigma-Aldrich, MO, USA) and Lightcycler 480II (Roche, Basel, Switzerland) were used for qRT-PCR, with a binding temperature of 59°C. The mRNA levels were normalized to reference gene *Hprt1* and reference gene stability was confirmed using R Statistical Software with the package ctrlGene [25], which uses the GeNorm algorithm. For the Calcium-Sensing receptor gene, *Casr*, the primer sequences are: TTCTCCAGAGAGGTGCCTTTCT (forward) and GGCACTCGCATCTGTCTCTCCA (reverse). For the Hypoxanthine phosphoribosyltransferase 1 gene, *Hprt1*, the primer sequences are: CCCAGCGTCGTGATTAGTGA (forward) and CCAAATCTTCAGCATAATGATTAGGTAT (reverse).

### RNA-sequencing and analysis

Next-Generation-Sequencing was performed at Department of Genomic Medicine, Rigshospitalet, Denmark. Total RNA was extracted using TRIzol (Sigma-Aldrich, St Louis, MO, USA) according to the manufacturer’s instructions and purified using Qiagen RNase-Free DNase Set (Qiagen, Hilden, Germany) on a QIAcube Connect (Qiagen, Hilden, Germany). RNA quality was examined using an Agilent Technologies 2100 Bioanalyzer. After quality control samples were available from 8 *ctrl1* rats, 4 *ctrl2* rats, 7 *PNX1* rats, 7 *PNX2* rats, 7 *Cina1* rats, and 10 *Cina2* rats. Library preparation was done using a TruSeq Stranded Total RNA Gold kit with dual adaptor indexing strategy; IDT for Illumina–TruSeq RNA UD Indexes (Illumina, San Diego, USA). The library was diluted and pooled to a concentration of 2 nM according to the procedure of “Denature and Dilute Libraries Guide of Illumina Novaseq 6000” and final loading concentration was 400 pM. Libraries were sequenced paired-end (2 x 151bp) to a sequencing depth of 40 M reads/sample by NovaSeq 6000 with SP Reagent Kit v1.5 (Illumina, San Diego, USA). Loading method was according to “NovaSeq 6000 Sequencing System Guide”. The fastQ data was generated by Novaseq automatically. RNA raw sequence reads were aligned to the reference genome (rn6) using STAR [26] (version 2.7.8a+galaxy0), uniquely mapped reads were counted using featureCounts [27] (version 2.0.1+galaxy1) and genes with less than 10 counts in total were excluded. Counts were then normalized for sequencing depth and RNA composition using the median of ratios method employed in DESeq2 [28] (version 1.30.1). Normalized counts were used for differential expression analysis using DESeq2 and the clusterProfiler [29] package (version 3.18.1) was used for gene ontology enrichment (enrichGO) and gene set enrichment analysis (GSEA) [30] with FDR-adjusted p-value at <0.05.

### Statistics

All statistical analyses were performed using the R Statistical Software (version 4.0.4) [31]. The circadian rhythmicity of *Casr* was analyzed by cosinor regression analysis [32]. For cosinor regression analysis, data were fitted to a linear model using the least squared method minimizing the residual sum of the squares:

$$Y(ZT)=MESOR+\beta ∙\cos\;\frac{2\pi ∙ZT}{24\;h}+\gamma ∙\sin\;\frac{2\pi ∙ZT}{24\;h}$$

ZT = zeitgebertime (h), MESOR = rhythm adjusted mean, β = A∙cos φ, γ = A∙sin φ, A = Amplitude, φ = Acrophase. The period was fixed at 24 h. Significant rhythm was found when the 95% confidence intervals of β or γ did not include “0”.

Welch’s two sample *t*-test or Mann-Whitney test was used for comparing two groups.

More than two groups were compared by Kruskal Wallis test with *post hoc* test after Dunn with Bonferroni adjustment. Results were classified as significant at p < 0.05. Values are presented as mean±SEM, except in S1 Table in S1 File presenting values as mean±SD.

### Ethics

The study was approved by the Danish Animal Inspectorate (Reference no. 2017-15-0201-01214) and executed in accordance with national guidelines for laboratory animals and in adherence to the NIH Guide for the Care and Use of Laboratory Animals. For the *Cinacalcet Chronotherapy* protocol, surgery was performed under Pentobarbital anesthesia, and all efforts were made to minimize suffering. Humane endpoints were behavioral signs of distress, reduced intake of food or water, and 20% weight loss. During the first 3 days after surgery, animals were monitored at least 3 times a day. After 3 days, animals are monitored at least once daily and weighted once weekly throughout the protocol. Rats were anesthetized with Pentobarbital (Nycomed-DAK, Denmark), 50 mg/ml, at dose of 1 μl/g rat and euthanized using a cardiac injection of Pentobarbital (Nycomed-DAK, Denmark), 200 mg/ml, at a dose of 1 ml.

## Results

### Parathyroid gene expression of *Casr* shows marked circadian rhythmicity in sHPT

**Fig 1:**
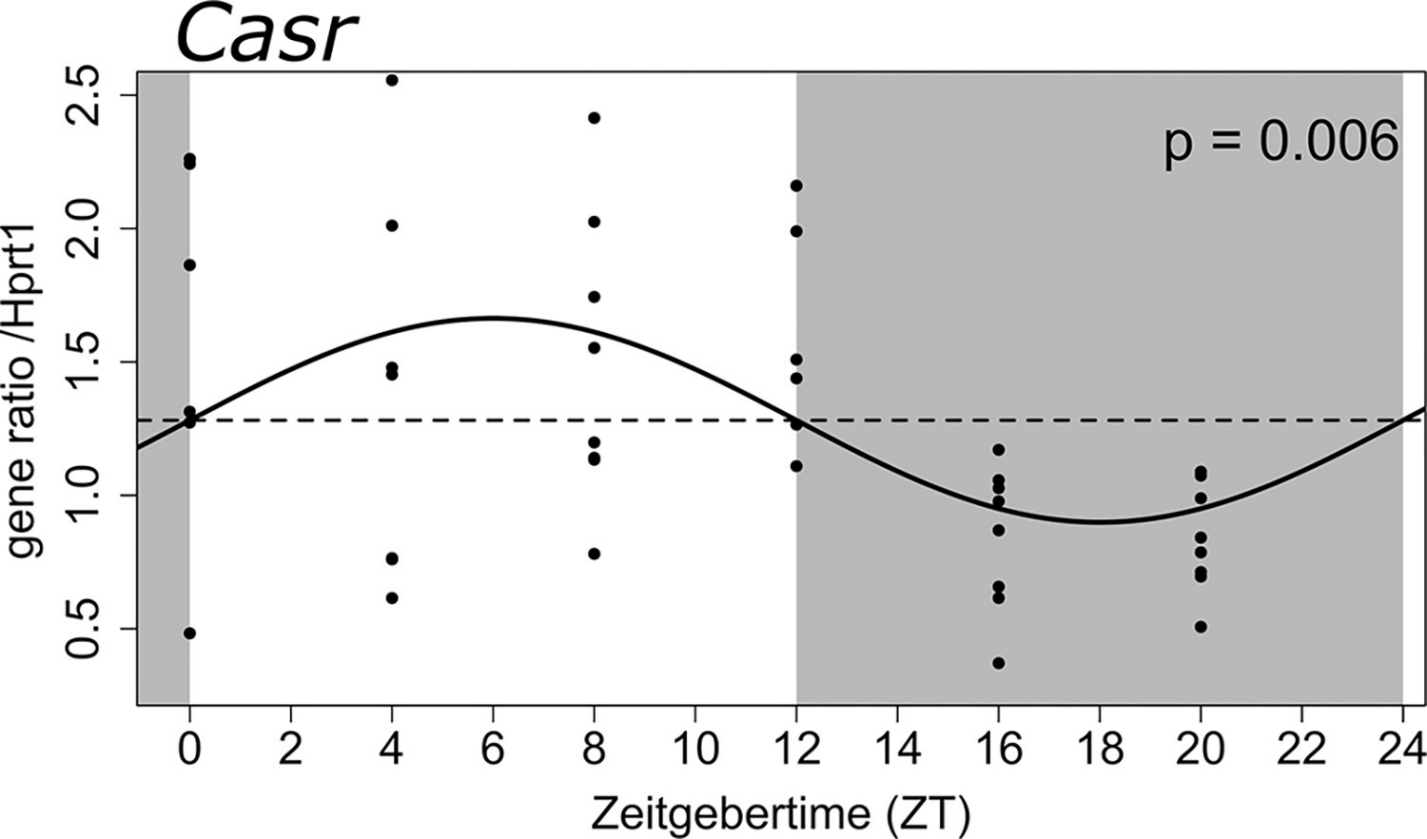
Parathyroid *Casr* gene expression is significantly rhythmic in secondary hyperparathyroidism. Expression profile of *Casr* gene over 24 h in parathyroid glands from 44 rats with secondary hyperparathyroidism. Rhythmicity was examined by cosinor regression analysis and showed significant 24 h rhythmicity of expression (p = 0.006). Dots represent one sample. Data are fitted by cosinor regression (solid line). Gray areas indicate dark period and white areas indicate light period. Zeitgebertime (ZT; “time-giver”) is hours since light onset. Gene expression was normalized to the expression of housekeeping gene *Hprt1*.

Parathyroid gene expression was examined at 4 h interval for 24 h of 44 rats with secondary hyperparathyroidism induced by 5/6 nephrectomy combined with a high phosphate diet. We found a significant circadian rhythmicity of the *Casr* gene expression (p = 0.006) with a clear decrease in expression during the active dark phase of the nocturnal rats (Fig 1).

### Chronotherapy–study design and biochemistry

**Fig 2:**
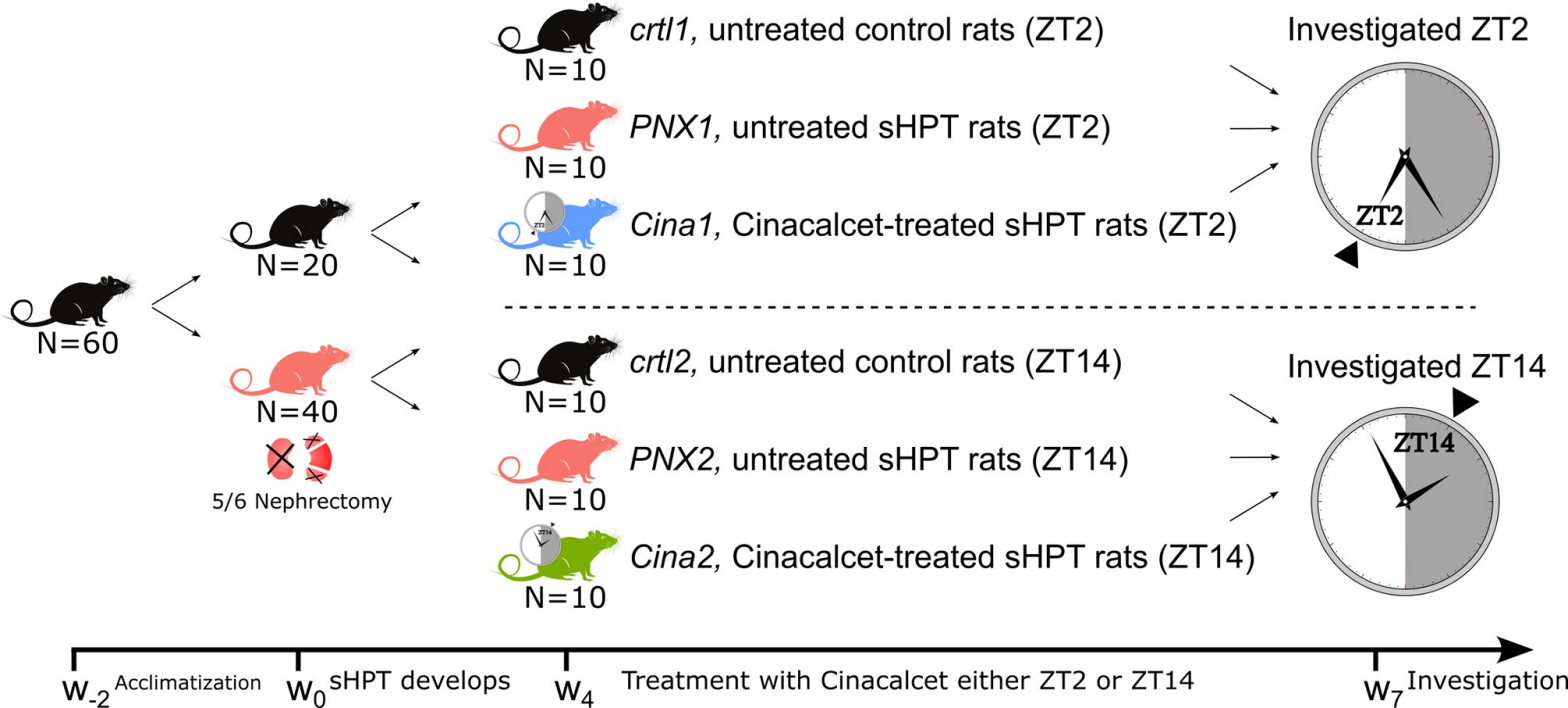
Chronotherapy study design. Schematic timeline of the Chronotherapy study. 60 rats were acclimatized for 14 days and, CKD was induced by 5/6 nephrectomy in 40 rats which were shifted to a high-phosphate diet to induce sHPT at w_0_. Four weeks later at w_4_, 20 rats with sHPT were randomized to receive Cinacalcet once daily at either ZT2 (N = 10) or ZT14 (N = 10). After three weeks of treatment at w_7_, Cinacalcet-treated rats were investigated at either ZT2 or ZT14, 24 h since last medication together with untreated normal and sHPT control rats (N = 10 of each). Gray areas indicate dark period and white areas indicate light period. ZT, Zeitgebertime (“time-giver”) is hours since light onset.

Having found diurnal variation in the expression of *Casr*, we next examined whether timing of Cinacalcet administration according to this rhythm, would increase the effect of treatment. To this end, we conducted a chronotherapy study using 60 rats as shown in Fig 2. The groups were comparable in weight before 5/6 nephrectomy was performed (Table 1: w_0_). There was a significant decrease in body weight and an increase in plasma urea, creatinine, and phosphate of the CKD groups compared to control groups four weeks post-nephrectomy (Table 1: w_4_). There was no significant difference in urea, creatinine, phosphate or calcium between CKD groups immediately before treatment was initiated (Table 1: w_4_) or after three weeks of Cinacalcet treatment at time of investigation, either ZT2 or ZT14 (Table 1: w_7_). Plasma PTH and FGF23 were significantly elevated in all CKD groups compared to both control groups with no significant difference between CKD groups before treatment (Table 1: w_4_). At time of investigation after 7 weeks of uremia, CASR immunostaining was significantly decreased in both untreated CKD groups compared to both control groups (S1 Fig in S1 File). Cinacalcet treatment maintained CASR expression at a similar level in the two treatment groups, with *Cina1* showing significant increase compared to both *PNX1* and *PNX2* and *Cina2* showing significant increase compared to *PNX2* (S1 Fig in S1 File). In both treatment groups, after three weeks of Cinacalcet treatment there was a significant reduction in FGF23 compared to untreated CKD group (Table 1: w_7_). Cinacalcet treatment at ZT2 (*Cina1*) resulted in a 55.1% decrease in PTH and treatment at ZT14 (*Cina2*) resulted in a 29.0% decrease in PTH compared to the respective untreated CKD control group measured at the same time point which was 24 h since last administration of Cinacalcet. Due to large biological variation, primarily in the untreated CKD groups, these differences did not reach statistical significance (Table 1: w_7_).

**Table 1:** Weight and plasma biochemistry.

			Morning (ZT2)			Evening (ZT14)	
		*ctrl1*	*PNX1*	*Cina1*	*ctrl2*	*PNX2*	*Cina2*
Weight (g)	w_0_	227±4	219±5	229±3	229±3	236±3	224±3
	w_4_	437±10	377±10^a,b^	390±7^a,b^	447±13	381±15^a,b^	390±7^a,b^
	w_7_	501±12	400±13^a^	411±10^a^	503±17	412±24^b^	388±18^b^
Urea (mmol/L)	w_4_	6.8±0.2	13.9±0.8^a,b^	13.1±0.7^a,b^	7.1±0.2	13.8±0.9^a,b^	14.2±0.7^a,b^
	w_7_	7.6±0.1	14.4±1.0^a^	14.5±1.8^a^	7.7±0.4	14.0±1.6^b^	12.4±0.6^b^
Creatinine (μmol/L)	w_4_	24±1	55±4^a,b^	59±5^a,b^	25±2	56±6^a,b^	58±4^a,b^
	w_7_	19±1	66±8^a^	62±8^a^	19±1	67±10^b^	61±6^b^
Phosphate (mmol/L)	w_4_	2.6±0.1	3.0±0.2^a,b^	3.0±0.2^a,b^	2.7±0.1	2.9±0.1^a,b^	3.2±0.1^a,b^
	w_7_	2.2±0.1	2.9±0.2^a^	2.9±0.2^a^	2.3±0.1	3.3±0.4^b^	2.9±0.2^b^
Calcium (mmol/L)	w_4_	2.7±0.0	2.6±0.1	2.7±0.1	2.8±0.0	2.6±0.1	2.6±0.1
	w_7_	2.7±0.0	2.4±0.1^a^	2.4±0.2^a^	2.7±0.0	2.3±0.15^b^	2.4±0.1^b^
Ionized Ca^2+^ (mmol/L)	w_7_	1.36±0.01	1.10±0.06^a^	1.15±0.03^a^	1.31±0.0	1.08±0.08^b^	1.15±0.04^b^
PTH (pg/ml)	w_4_	70±19	1629±337^a,b^	1498±321^a,b^	86±19	1511±392^a,b^	1978±316^a,b^
	w_7_	230±85	5076±1740^a^	2279±447^a^	215±36	3756±911^b^	2667±594^b^
FGF23 (pg/ml)	w_4_	520±29	2258±378^a,b^	2191±325^a,b^	502±81	2075±197^a,b^	1903±147^a,b^
	w_7_	445±48	2598±560^a^	1465±252^a,c^	523±57	1939±315^b^	1131±170^b,d^

w_0_ denotes time of nephrectomy. Weights at w_0_ are compared between all groups.

w_4_ denotes 4 weeks post nephrectomy, before start of treatment, samples collected ZT3-5. Comparisons at w_4_ are made between all groups.

w_7_ denotes 7 weeks post nephrectomy, at three weeks of treatment, samples collected ZT2 (*Cina1*, *PNX1* and *ctrl1*) or ZT14 (*Cina2*, *PNX2* and *ctrl2*) which is 24 h since last medication. Comparisons at w_7_ are made between samples collected at the same ZT.

^a^ p < 0.05 compared to *ctrl1;* ^b^ p < 0.05 compared to *ctrl2;* ^c^ p < 0.05 compared to *PNX1;* ^d^ p < 0.05 compared to *PNX2*.

Values are means ± SEM

### Bone micro-CT in CKD

Hyperparathyroid bone disease is a serious complication of sHPT in patients with CKD. μCT of left mid-femur revealed significantly decreased cortical thickness and increased porosity of CKD groups compared to normal control groups, except between *Cina1* and *ctrl1* (S1 Table and S2A Fig in S1 File). There was no significant difference in mid-diaphyseal cortical porosity between CKD groups. The bone volume fraction of the trabecular bone seemed to be increased in all CKD groups, although this did not reach the level of statistical significance (S1 Table and S2B Fig in S1 File). Within all CKD samples, cortical bone porosity was significantly associated with increasing trabecular bone volume (p<10^−6^, not shown) and with increasing plasma PTH (p<10^−7^, S2D Fig in S1 File).

### Aortic calcification in CKD

Previous reports have demonstrated protective effect of Cinacalcet treatment on vascular calcification [33]. In the control groups, aortic calcium content was 0.53±0.02 μg/mg (*ctrl1*) and 0.54±0.03 μg/mg (*ctrl2*). Vascular calcification was significantly increased in the CKD groups *PNX1* (0.81±0.05 μg/mg), *PNX2* (0.94±0.11 μg/mg), and *Cina1* (0.80±0.06 μg/mg) compared to normal controls, whereas aortic calcification in *Cina2* (0.73±0.04 μg/mg) was not significantly elevated compared to normal controls (S2C Fig in S1 File). The aortic calcification did not differ between CKD groups. Thus, the increased aortic calcification in the CKD groups was not significantly attenuated by the Cinacalcet treatment.

### Cinacalcet administered early in the inactive phase markedly inhibits proliferation of the parathyroid gland

**Fig 3:**
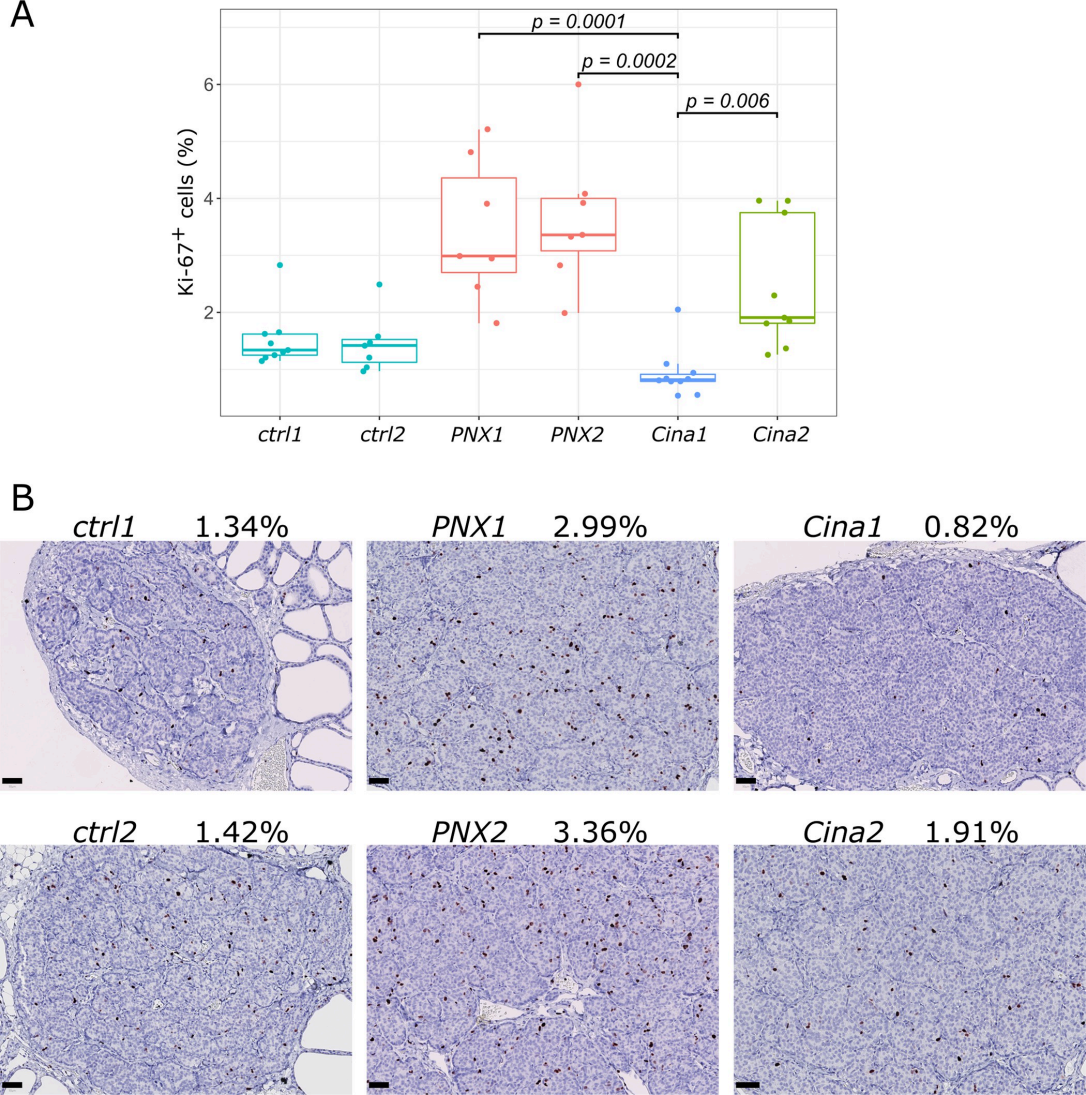
Cinacalcet administered early in the inactive phase markedly decrease parathyroid Ki-67 index. (A) Ki-67 expression in parathyroid glands of rats with CKD-induced sHPT treated with Cinacalcet either early in the inactive light phase (*Cina1*; N = 10) or early in the active dark phase (*Cina2*; N = 9) compared to untreated rats with sHPT investigated at similar time points (*PNX1*; N = 7 and *PNX2*; N = 7, respective) and to normal rats investigated at similar time points (*ctrl1*; N = 9 and *ctrl2*; N = 7, respective). All groups were compared by Kruskal Wallis test with *post hoc* test after Dunn with Bonferroni adjustment showing significant decreased Ki-67 labeling index of *Cina1* compared to *Cina2* (p = 0.006) and the untreated CKD groups (p = 0.0001 and p = 0.0002, respectively). (B) For each group, the median Ki-67 immunostained parathyroid sample closest to the group mean is shown. Each dot represents one sample. Scale bars measures 50μm.

Parathyroid proliferation was assessed by Ki-67 immunostaining of thyroparathyroid tissue sections obtained at either ZT2 or ZT14, which was 24 h post last treatment for Cinacalcet treated groups and expressed as the fraction of Ki-67 positive cells in the parathyroid gland (Fig 3). There was comparable Ki-67 immunostaining of the parathyroid glands between normal controls at the two time points (*ctrl1* vs. *ctrl2*: n.s.) and also between the untreated CKD control groups at the two time points (*PNX1* vs. *PNX2*: n.s.). The untreated CKD groups had significantly higher Ki-67 labeling index than the control groups (*PNX1* vs. *ctrl1*: p = 0.0007) and (*PNX2* vs. *ctrl2*: p = 0.0012). The percentage of Ki-67 positive cells in the parathyroid glands of all groups across time points are shown in Fig 3A and the Ki-67 staining of the median sample closest to the mean from each group is presented in Fig 3B. We found a strikingly decreased Ki-67 labeling index when Cinacalcet was administered early in the inactive phase at ZT2 (*Cina1*) as compared to when Cinacalcet was administered early in the active phase at ZT14 (*Cina1* vs. *Cina2*: 0.92±0.14% vs. 2.46±0.37%, p = 0.006) and when compared to the untreated CKD groups at ZT2 (*Cina1* vs. *PNX1*: 0.92±0.14% vs. 3.45±0.47%, p = 0.0002) and at ZT14 (*Cina1* vs. *PNX2*: 0.92±0.14% vs. 4.15±0.47%, p = 0.0001), respectively.

### RNAseq analysis of the parathyroid glands in sHPT

**Fig 4:**
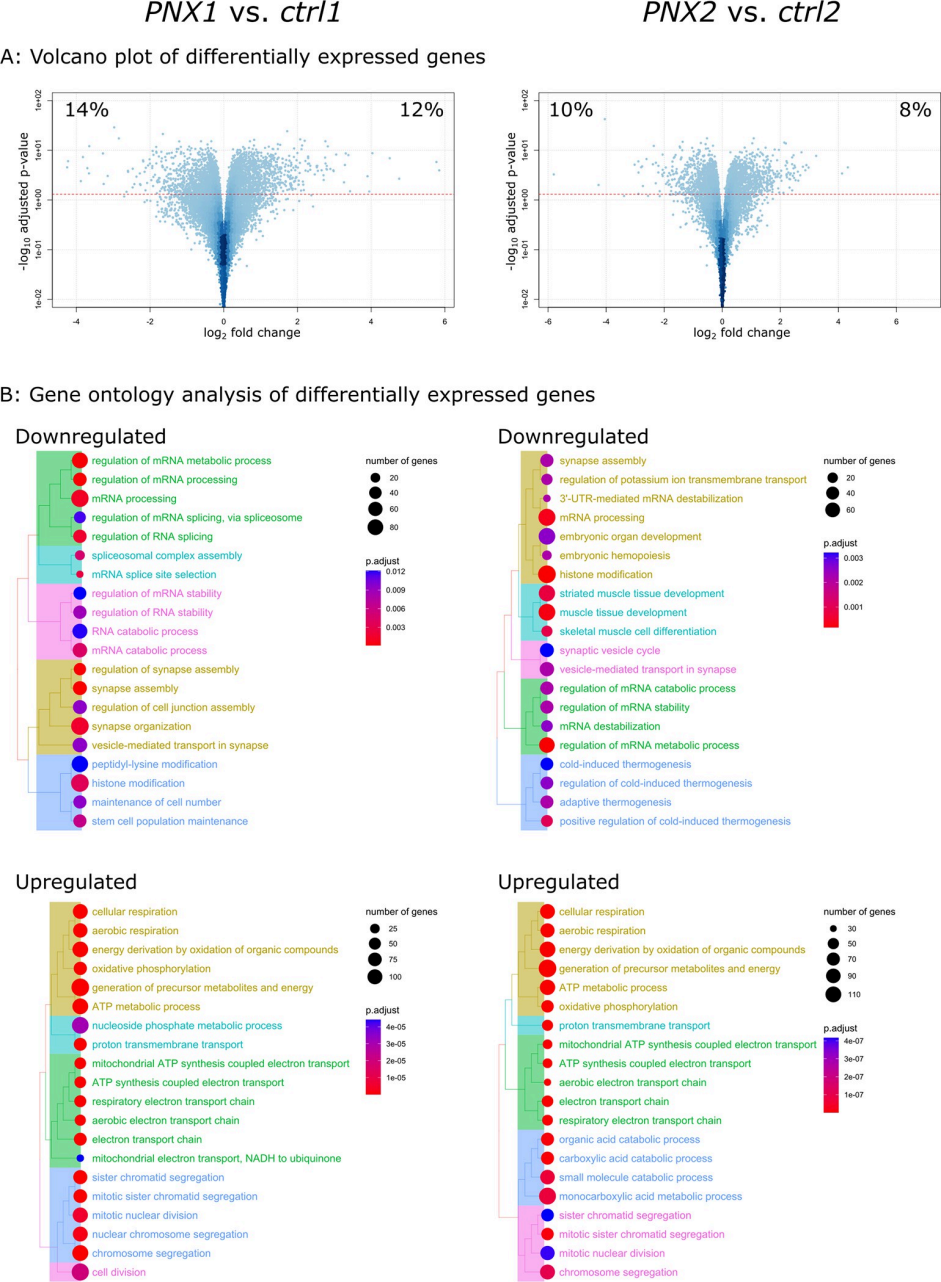
Transcriptional regulation is downregulated, and oxidative phosphorylation is upregulated in parathyroid glands in sHPT. The transcriptomes of parathyroid glands from untreated CKD groups *PNX1* (N = 7) and *PNX2* (N = 7) were compared to glands from normal rats investigated at similar time points: *ctrl1* (N = 8) and *ctrl2* (N = 4), respectively. (A) Comparing *PNX1* to *ctrl1* 14% of all expressed genes were downregulated and 10% upregulated, which was the case for 10% and 8%, respectively, comparing *PNX2* to *ctrl2*. (B) Downregulated genes at both investigated time points were significantly enriched in terms of *mRNA processing* and *histone modification* by gene ontology analysis and upregulated genes of both comparisons were enriched in terms related to *ATP synthesis/Oxidative phosphorylation* and *mitotic nuclear division*. Dashed line in (A) indicates adjusted p-value of <0.05. For all p-values and further details, please see S2 Table in S1 File.

To investigate the mechanisms behind the marked difference in proliferation derived from timing of Cinacalcet administration, RNAseq was performed on micro-dissected and snap-frozen parathyroid samples from all groups. First, we compared the transcriptome of hyperplastic parathyroid glands in sHPT at either of the two time points: *PNX1* and *PNX2*, to that of normal parathyroid glands harvested at the same time point; *ctrl1* and *ctrl2*, respectively. For both comparisons we found that the transcriptomes of hyperplastic parathyroid glands were well separated from those of normal glands by PCA plot (S3A Fig in S1 File) and that a large percentage of all expressed genes were significantly differentially expressed (26% for *PNX1 vs*. *ctrl1* and 18% for *PNX2 vs*. *ctrl2*, Fig 4A). In both cases, gene ontology analysis of the significantly downregulated genes showed significant enrichment in terms of *mRNA processing* and *histone modification* (adjusted p-value<0.003, Fig 4B) and these terms were also enriched in the overlapping downregulated genes between the two comparisons (S3C Fig in S1 File). Furthermore, gene ontology analysis of the significantly upregulated genes of both comparisons, *PNX1 vs*. *ctrl1* and *PNX2 vs*. *ctrl2*, showed significant enrichment in multiple terms related to *ATP synthesis/Oxidative phosphorylation* and *mitotic nuclear division* (adjusted p-value<10^−5^, Fig 4B) and these terms were also enriched in the overlapping upregulated genes (S3D Fig in S1 File). Non-overlapping differentially expressed genes showed no significant enrichments except for the upregulated genes only in the *PNX2 vs*. *ctrl2* comparison, which were enriched in genes related to *fatty acid beta-oxidation* (S3D Fig in S1 File).

**Fig 5:**
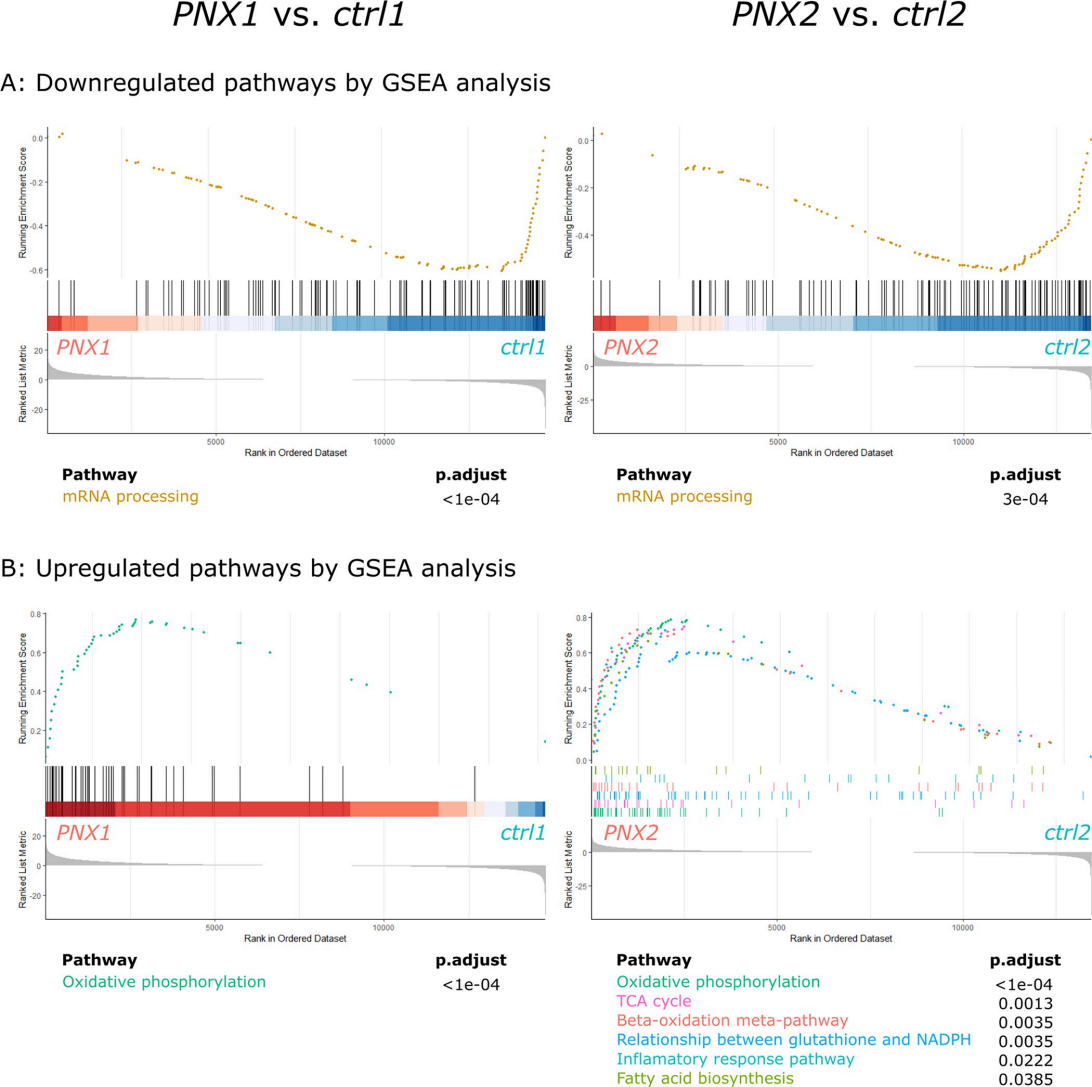
Pathways of mRNA processing are downregulated, and pathways of oxidative phosphorylation are upregulated in parathyroid glands in sHPT. The transcriptomes of parathyroid glands from untreated CKD groups *PNX1* (N = 7) and *PNX2* (N = 7) were compared to glands from normal rats investigated at similar time points: *ctrl1* (N = 8) and *ctrl2* (N = 4), respectively. (A) Gene Set Enrichment Analysis (GSEA) found significant downregulation of one pathway: *mRNA processing (WP529)* and (B) upregulation of the pathway of *Oxidative phosphorylation (WP1283)* in both comparisons, whereas in the *PNX2 vs*. *ctrl2* comparison also pathways of *TCA cycle (WP347)*, *Beta-oxidation meta-pathway (WP372)*, *Fatty acid biosynthesis (WP504)*, *Relationship between glutathione and NADPH (WP2562)* and *Inflammatory response pathway (WP40)* were upregulated. For all normalized enrichment scores (NES), p-values and further details, please see S2 Table in S1 File.

Gene Set Enrichment Analysis (GSEA) compares two conditions by ranking genes of known pathways by expression, to test for significant up- or downregulation between conditions. GSEA found downregulation of the pathway: *mRNA processing (WP529)* in both comparisons *PNX1 vs*. *ctrl1* and *PNX2 vs*. *ctrl2* (adjusted p<0.0003, Fig 5A) and GSEA found upregulated pathway of *Oxidative phosphorylation (WP1283)* in both comparisons (adjusted p-value<10^−6^), whereas in the *PNX2 vs*. *ctrl2* comparison also pathways of *TCA cycle (WP347)*, *Beta-oxidation meta-pathway (WP372)*, *Fatty acid biosynthesis (WP504)*, *Relationship between glutathione and NADPH (WP2562)* and *Inflammatory response pathway (WP40)* were upregulated (adjusted p<0.04) (Fig 5B). For all p-values and schematic overview of significant changes, please see S2 and S3 Tables in S1 File, respectively.

Leading-edge genes are the genes contributing most to the signal in GSEA analysis. Leading-edge genes of the downregulated *mRNA processing* pathway showed widespread overlap between the two time points and included the majority of Serine/Arginine rich proteins involved in mRNA splicing (S4 Fig in S1 File). Likewise, leading-edge genes of the upregulated *Oxidative phosphorylation* pathway showed extensive overlap between the two time points and included genes encoding proteins of the mitochondrial respiratory chain complex I converting NADH to NAD^+^ and genes encoding proteins of the ATP synthase complex converting ADP into ATP (S5 Fig in S1 File).

Taken together, RNAseq analysis of the parathyroid glands in two independent comparisons, morning and evening, showed a concordant decrease in the *mRNA processing* pathway and an increase in the pathway of mitochondrial *Oxidative phosphorylation* in sHPT.

### RNAseq analysis of parathyroids obtained from rats with sHPT treated with Cinacalcet reveals downregulation of genes involved in proliferation and upregulation of pathways of energy metabolism, but only when Cinacalcet is administered in the morning, *Cina1*

**Fig 6:**
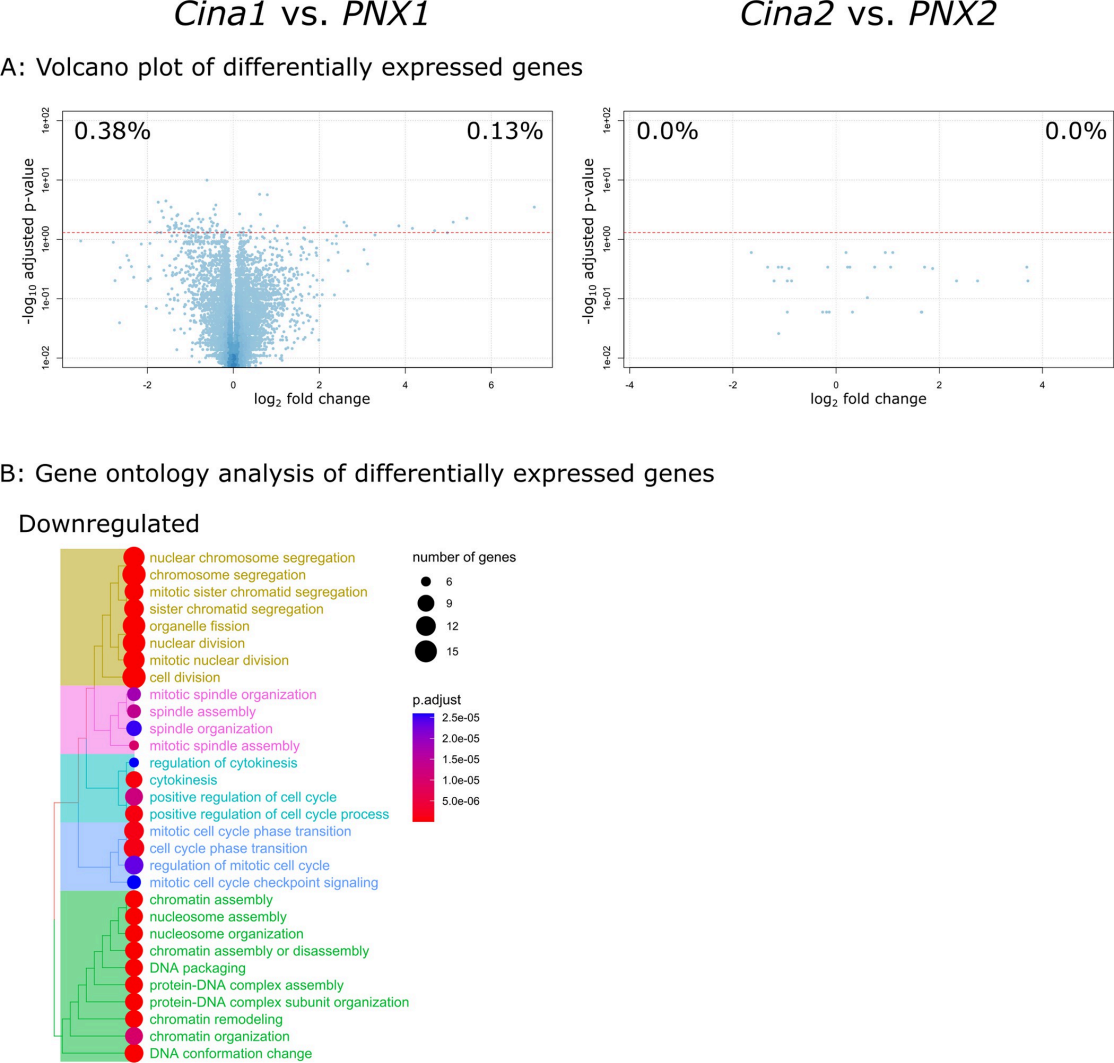
Cinacalcet administered before the inactive phase leads to downregulation of genes involved in mitotic nuclear division. The transcriptomes of parathyroid glands from Cinacalcet treated CKD groups *Cina1* (N = 7) and *Cina2* (N = 10) were compared to glands from untreated CKD rats investigated at similar time points: *PNX1* (N = 6) and *PNX2* (N = 7), respectively. (A) Comparing *Cina1* to *PNX1*, 67 genes were downregulated and 23 upregulated. No genes were differentially expressed comparing *Cina2* to *PNX2*. (B) The downregulated genes of *Cina1* were significantly enriched in multiple terms involved in *chromatin organization*, *mitotic cell cycle checkpoint transition* and *mitotic nuclear division* by gene ontology analysis, whereas upregulated genes showed no significant enriched terms. For all normalized enrichment scores (NES), p-values and further details, please see S2 Table in S1 File.

Transcriptomes of parathyroid samples from the two treatment groups (*Cina1* and *Cina2*) were compared to the corresponding untreated CKD control groups (*PNX1* and *PNX2*, respectively). When comparing the transcriptome of *Cina1* to that of *PNX1* we found 67 significantly downregulated genes and 23 significantly upregulated genes by differential expression analysis using an adjusted p-value of <0.05. Surprisingly, we found no significantly differentially expressed genes when comparing the transcriptome of *Cina2* to that of *PNX2* (Fig 6A). Gene ontology analysis of significantly downregulated genes in *Cina1* were enriched in multiple terms involved in *chromatin organization*, *mitotic cell cycle checkpoint transition* and *mitotic nuclear division* (Fig 6B). Upregulated genes in *Cina1* showed no significant enrichment of gene ontology terms.

**Fig 7:**
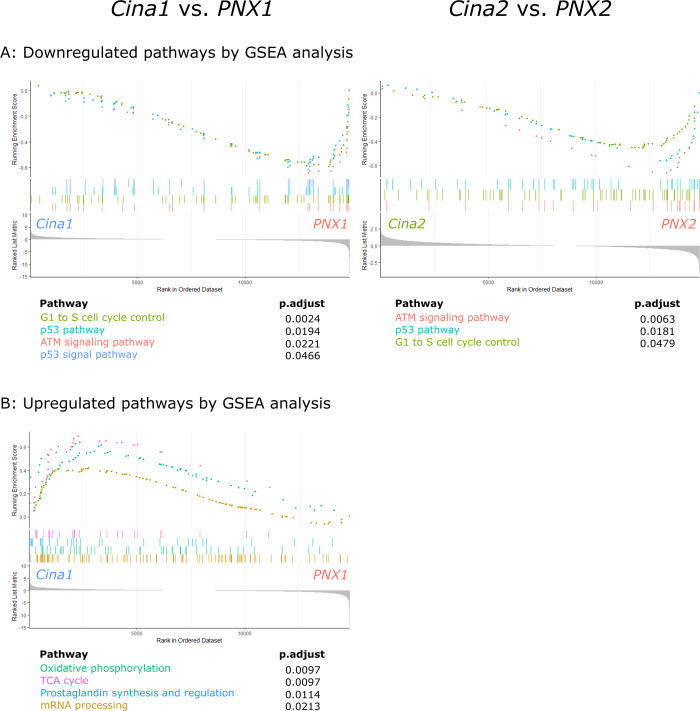
Cinacalcet administered before the inactive phase upregulate pathways of energy metabolism. The transcriptomes of parathyroid glands from Cinacalcet treated CKD groups *Cina1* (N = 7) and *Cina2* (N = 10) were compared to glands from untreated CKD rats investigated at similar time points: *PNX1* (N = 6) and *PNX2* (N = 7), respectively. (A) Gene Set Enrichment Analysis (GSEA) found in both Cinacalcet treated groups a significant downregulation of the pathways: *ATM signaling pathway (WP654)*, *p53 pathway (WP655)* and *G1 to S cell cycle control (WP348)* whereas only Cinacalcet administered in the early inactive phase (*Cina1*) resulted in significant upregulation of the pathways *Oxidative phosphorylation (WP1283)* and *TCA cycle (WP347)*, *mRNA processing (WP529)* and *Prostaglandin synthesis and regulation (WP303)* (B). For all normalized enrichment scores (NES), p-values and further details, please see S2 Table in S1 File.

GSEA found that the two Cinacalcet treated groups, when compared to their respective untreated CKD control groups, exhibited significant downregulation of the exact same pathways involved in the *ATM signaling pathway (WP654)*, *p53 pathway (WP655)* and *G1 to S cell cycle control (WP348)* as shown in Fig 7A. In addition, GSEA of *Cina1* vs. *PNX1* also showed downregulation of the closely associated pathway: *p53 signal pathway (WP656)*. When comparing the leading-edge genes of downregulated pathways from both comparisons, we found large overlaps ranging from 37.5% to 61.1% of all leading-edge genes. GSEA analysis showed no upregulated pathways when comparing *Cina2* to *PNX2*, whereas four pathways were significantly upregulated in comparing *Cina1* to *PNX1* (Fig 7B). Of these, two pathways were involved in energy metabolism: *Oxidative phosphorylation (WP1283)* and *TCA cycle (WP347)*, one was involved in *mRNA processing (WP529)* and one in *Prostaglandin synthesis and regulation (WP303)*. Leading-edge genes of the upregulated *mRNA processing* pathway in *Cina1* compared to *PNX1* showed large overlap with leading-edge genes of both comparisons between the *PNX* groups to *ctrl* groups in which this same pathway was significantly downregulated (S4 Fig in S1 File). Leading-edge genes of the upregulated *Oxidative phosphorylation* pathway in *Cina1* compared to *PNX1* showed substantial overlap with leading-edge genes of both comparisons between the *PNX* groups to *ctrl* groups in which this same pathway was also significantly upregulated (S5 Fig in S1 File).

Taken together, results of the RNAseq analysis showed Cinacalcet-induced downregulation of genes involved in proliferation and upregulation of energy metabolism pathways, but only when Cinacalcet was administered in the morning, *Cina1*. Furthermore, Cinacalcet treatment resulted in downregulation of cellular stress pathways at both time points of administration.

## Discussion

The present study examined the possible benefit of timing Cinacalcet treatment to the diurnal rhythm of its target; the calcium-sensing receptor. We found that the parathyroid expression of the *Casr* gene was subjected to diurnal rhythmicity and was downregulated in the active period of the uremic rats. We conducted a treatment study administering Cinacalcet when the expression of *Casr* is high, early in the inactive period (*Cina1*), compared to the conventional administration time early in the active period (*Cina2*). Ki-67 is a widely used and recognized proliferation marker present during all active phases of the cell cycle, but absent in quiescent, non-proliferative cells [34, 35]. Parathyroid proliferation assessed by Ki-67 immunostaining showed a striking reduction in the number of Ki-67^+^ cells in the *Cina1* group, which was significantly lower than that of the *Cina2* group and the two untreated uremic groups using a conservative Bonferroni adjustment for multiple group comparison. Remarkably, 9 out of 10 parathyroid samples from the uremic *Cina1* group showed a reduction in the Ki-67 labeling index to a level that was below the mean of the two normal control groups (*ctrl1* and *ctrl2*).

It is not possible to directly compare PTH levels of the two Cinacalcet treated groups as PTH exhibits a marked diurnal variation, peaking in the inactive period [4] leading to a falsely elevated level in *Cina1* measured at ZT2 compared to *Cina2* measured at ZT14. Comparing the two *Cina* groups to *PNX* groups investigated at the same time point, it seems that even 24 h after last administration, Cinacalcet timed for the inactive phase led to a greater reduction in PTH compared to the untreated CKD group.

To unravel the mechanism behind the marked effect on parathyroid proliferation by timing of Cinacalcet administration, we investigated the transcriptomes of *Cina1* and *Cina2* and compared them to that of untreated CKD controls, *PNX1* and *PNX2*, respectively. By GSEA analysis, we found that treatment at both time points led to a significant downregulation of the pathway: *G1 to S cell cycle control* and of the two related cellular stress pathways: *ATM pathway* and *p53 pathway*. Administering Cinacalcet early in the inactive period (*Cina1*) led to a significant downregulation of 67 genes which were enriched in gene ontology terms of *mitotic nuclear division*, indicating that Cinacalcet treatment at this administration time point inhibited the increased mitotic activity of parathyroid cells in sHPT.

GSEA analysis showed that the *mRNA processing* pathway was downregulated in both CKD groups (*PNX1* and *PNX2*) compared to controls (*ctrl1* and *ctrl2*) with large overlaps of leading-edge genes. This pathway was upregulated in *Cina1* compared to the time-matched untreated CKD group; *PNX1*. These results indicate that at this time of administration, Cinacalcet treatment counteracts the decreased mRNA processing of hyperplastic parathyroid glands compared to normal glands.

In sHPT, parathyroid glands greatly increase PTH biosynthesis and secretion, which requires increases in energy metabolism, as evident from the comparisons of *PNX* groups against *ctrl* groups, which both showed a marked upregulation of the *Oxidative phosphorylation* pathway. This adaptive response seemed to be further enhanced by Cinacalcet treatment in *Cina1* animals since pathways of *Oxidative phosphorylation* and *TCA cycle* was further upregulated comparing this group to the untreated CKD group, *PNX1*. This is very interesting in the light of our recent publication, which found in transgenic mice, that parathyroid glands with marked downregulation of the *Oxidative phosphorylation*/*Electron transport chain* and *TCA cycle* pathways had an insufficient compensatory PTH response to uremia leading to hypocalcemia and marked increase in Ki-67^+^ and PCNA^+^ cells [5]. Collectively, our results point to a pivotal and complex role of sufficient energy metabolism in controlling the proliferation of parathyroid chief cells in sHPT.

Studies in humans and rats have shown that vascular smooth muscle cells express CASR suggesting protective effects of Cinacalcet on vascular calcification through mechanism depending on the CASR [36, 37]. A study using 5/6 nephrectomized rats found significantly reduced von Kossa staining in tunica media in aorta after 12 weeks of Calcimimetic treatment compared to vehicle treatment [36]. In the current study, 3 weeks of Cinacalcet treatment had no protective effect on aortic calcification. We suspect that a longer treatment period may be necessary for a significant effect to manifest on this outcome. Few studies have investigated the effects of cinacalcet treatment on bone parameters. One study in nephrectomized rats found direct actions of calcimimetics on bone cell activity and bone formation rate [38] and this was supported in a *post hoc* analysis of an observational study [39]. In the EVOLVE trial, Cinacalcet treatment did not reduce bone fractures significantly in the intent-to-treat analysis, whereas a lag censoring analysis revealed a significant reduction in time to first fracture [11]. The current study did not find any significant protective effect of 3 weeks of Cinacalcet treatment in CKD in terms of maintaining cortical bone mass or on other bone microstructural properties as examined by μCT.

Animal studies of sHPT have several limitations in translating to the human setting and it is not known whether similar rhythmic *Casr* expression is found in parathyroid cells of human patients with sHPT. Moreover, current animal models of sHPT do not induce parathyroid adenoma formation as is most often present at the time of Cinacalcet initiation in human patients with sHPT. Hence, the results of the current investigation will need to be confirmed in a human study. If a beneficial effect can be demonstrated in humans, altering the administration time would be a very easy and cost-efficient adjustment to improve the treatment of sHPT. Based on the results of the current study, such an effect may be truly substantial.

In conclusion, the current study reveals a striking anti-proliferative effect of utilizing the principles of chronotherapy to optimize the administration time of Cinacalcet in a rat model of sHPT. We found significant diurnal rhythmicity of the *Casr* gene, peaking in the inactive phase, and by timing Cinacalcet treatment to this peak we achieved a marked inhibition of the severely increased parathyroid proliferation in sHPT to a level below the proliferative rate of normal parathyroid cells not subjected to uremia. We found that Cinacalcet downregulated pathways of cell cycle transitioning and cellular stress response at both administration times. However, only when administration of Cinacalcet was timed to the *Casr* peak did it counteract the downregulated mRNA processing pathways found in sHPT and further upregulated the pathways of energy metabolism, which may be essential to avoid excessive parathyroid proliferation in sHPT. The present results therefore indicate a potential significant benefit of using chronotherapy in the Cinacalcet treatment of sHPT.

## Supporting information

S1 File (PDF)
