# Supplementary material for: Chronotherapy with Cinacalcet has a striking effect on inhibition of parathyroid gland proliferation in rats with secondary hyperparathyroidism
Source: PLoS One. 2025 Jan 6;20(1):e0316675. doi: 10.1371/journal.pone.0316675 (PMC11703009; doi:10.1371/journal.pone.0316675)
Supplement: S1 File — (PDF) [file pone.0316675.s001.pdf]

Supporting information

SUPPLEMENTARY METHODS

Study design supplementary information

Age-matched rats were purchased at Charles River (Germany), and all animals were included in the predefined protocols. Animals were randomized for timepoints of investigation and intervention group by cage using online random number generator. No animals were excluded and no data points during the analysis were excluded. Outliers are included in analysis and figures. For the protocol "*Casr gene expression over 24 h*" sample size at each timepoint was based on SD and cosinor analyses from previous experiments investigating diurnal rhythms in the rat. For the protocol "*Cinacalcet Chronotherapy*" sample size of 10 animals per group was determined using SD from our previous Ki-67 staining data on rat parathyroid glands as this is the primary outcome of the protocol. During the study, order of treatment and measurement were performed randomly. For final outcome assessment, all operators were blinded to animal groups during the steps of harvesting organs, preparation of tissues, µCT measurements, aortic calcification measurements, RNAseq preparation, tissue staining and Ki-67 quantification.

SUPPLEMENTARY FIGURES

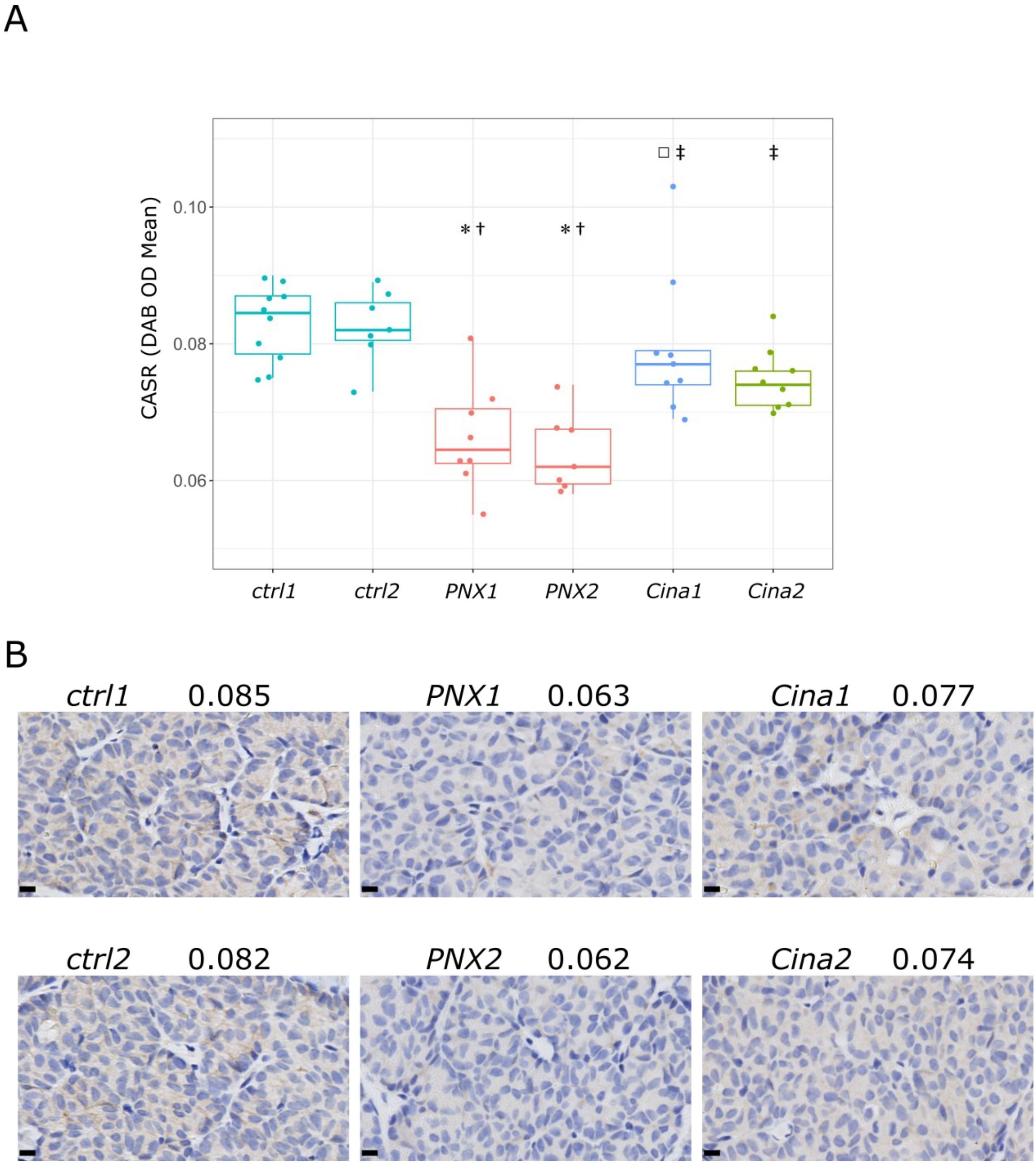

Fig S1: Cinacalcet treatment maintains parathyroid CASR expression

(A) CASR expression in parathyroid glands of rats with CKD-induced sHPT treated with Cinacalcet either early in the inactive light phase (*Cina1*; N=9) or early in the active dark phase (*Cina2*; N=9) compared to untreated rats with sHPT investigated at similar time points (*PNX1*; N=8 and *PNX2*; N=7, respective) and to

normal rats investigated at similar time points (*ctrl1*; N=10 and *ctrl2*; N=7, respective). (B) For each group, the median CASR immunostained parathyroid sample closest to the group mean is shown. Each dot represents one sample. * denotes $p < 0.05$ compared to *ctrl1*. † denotes $p < 0.05$ compared to *ctrl2*. □ denotes $p < 0.05$ compared to *PNX1.* ‡ denotes $p < 0.05$ compared to *PNX2.* Scale bars measures 10µm.

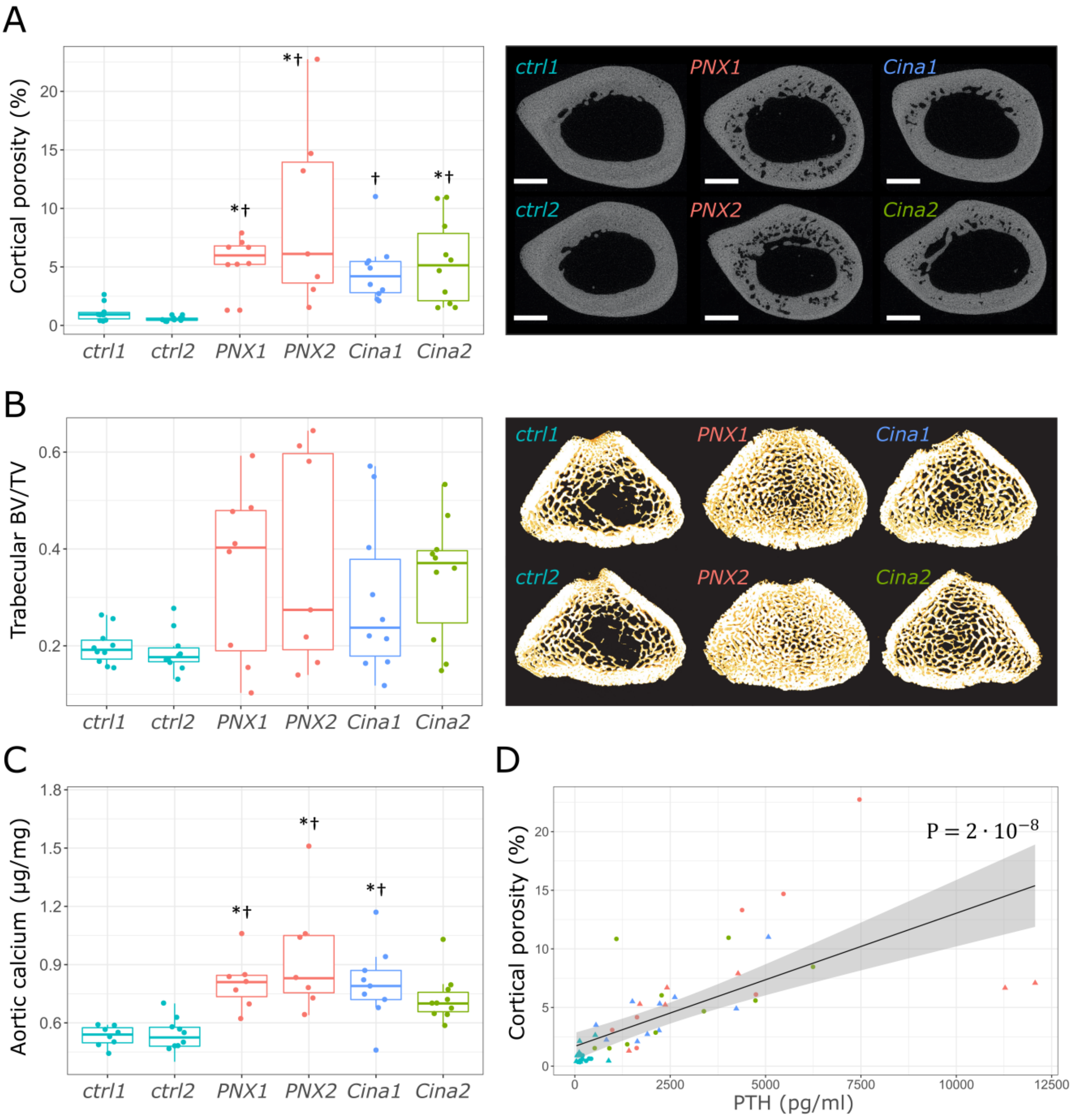

Fig S2: Femoral bone micro properties and aortic calcium content

The mid-diaphysis and distal metaphysis of the left femora were scanned in a desktop µCT and calcium content measured in the ascending aorta. (A) Porosity of mid-diaphysis cortical bone was significantly increased in all CKD groups compared to normal control groups except for the comparison between *Cina1* and *ctrl1*. There were no significant changes between CKD groups. (B) BV/TV of distal metaphysis trabecular bone showed a tendency towards increased bone deposition in CKD groups. (C) Aortic calcium content was

significantly increased in CKD groups except for *Cina2*. (D) Cortical porosity was strongly associated with PTH levels across all groups indicated by colors and shapes (gray = *ctrl*, red = *PNX*, blue = *Cina1*, green = *Cina2*, triangles = ZT2, circles = ZT14). Regression line is shown as a solid line with 95% CI (gray area). In (A) and (B) the median sample closest to the group mean is shown for each group. * denotes $p < 0.05$ compared to *ctrl1*. † denotes $p < 0.05$ compared to *ctrl2*. In (A) white bars measures 1mm.

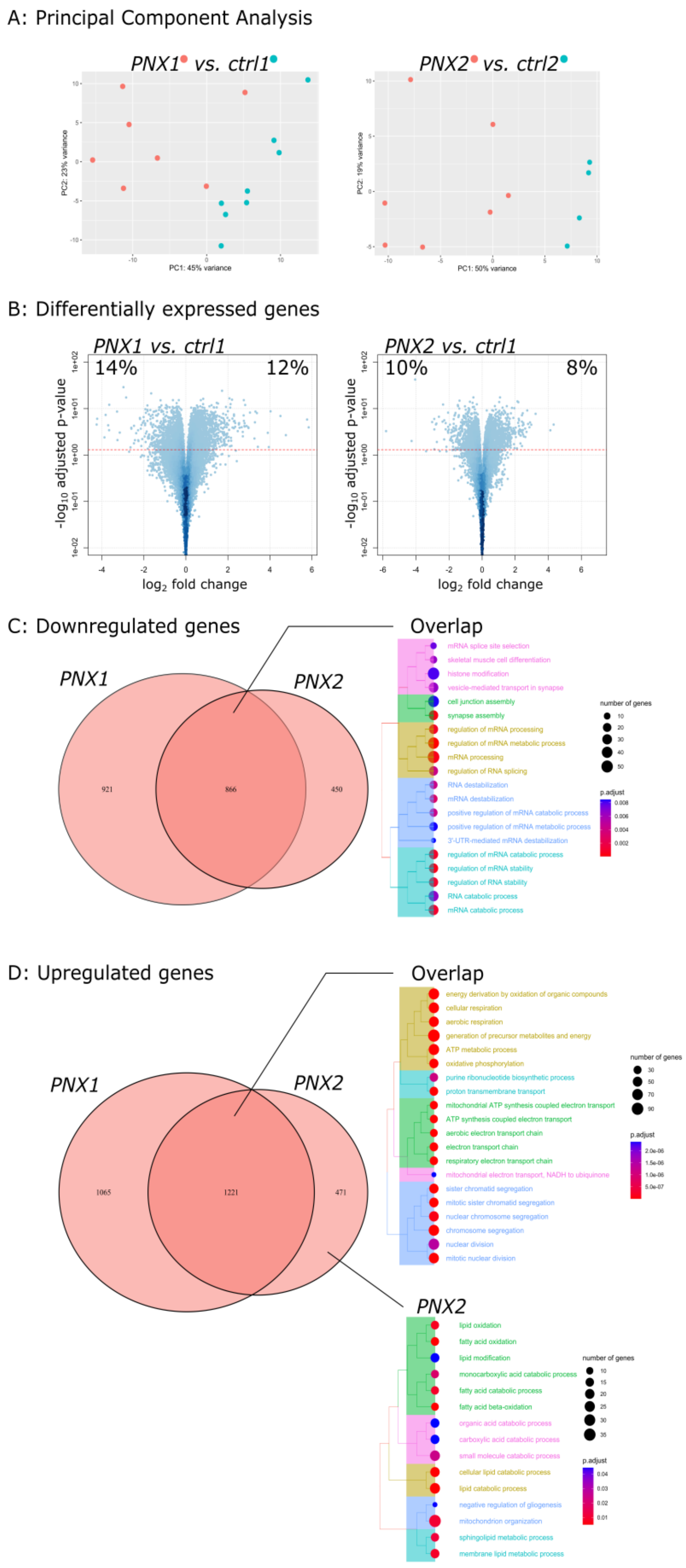
A: Principal Component Analysis
PNX1 vs. ctrl1
PC1: 45% variance
PC2: 23% variance
PNX2 vs. ctrl2
PC1: 50% variance
PC2: 19% variance
B: Differentially expressed genes
PNX1 vs. ctrl1
14%
12%
PNX2 vs. ctrl1
10%
8%
-log10 adjusted p-value
log2 fold change
C: Downregulated genes
PNX1
PNX2
921
866
450
Overlap
mRNA splice site selection
skeletal muscle cell differentiation
histone modification
vesicle-mediated transport in synapse
cell junction assembly
synapse assembly
regulation of mRNA processing
regulation of mRNA metabolic process
mRNA processing
regulation of RNA splicing
RNA destabilization
mRNA destabilization
positive regulation of mRNA catabolic process
positive regulation of mRNA metabolic process
3'-UTR-mediated mRNA destabilization
regulation of mRNA catabolic process
regulation of mRNA stability
regulation of RNA stability
RNA catabolic process
mRNA catabolic process
number of genes
p.adjust
D: Upregulated genes
PNX1
PNX2
1065
1221
471
Overlap
energy derivation by oxidation of organic compounds
cellular respiration
aerobic respiration
generation of precursor metabolites and energy
ATP metabolic process
oxidative phosphorylation
purine ribonucleotide biosynthetic process
proton transmembrane transport
mitochondrial ATP synthesis coupled electron transport
ATP synthesis coupled electron transport
aerobic electron transport chain
electron transport chain
respiratory electron transport chain
mitochondrial electron transport, NADH to ubiquinone
sister chromatid segregation
mitotic sister chromatid segregation
nuclear chromosome segregation
chromosome segregation
nuclear division
mitotic nuclear division
number of genes
p.adjust
PNX2
lipid oxidation
fatty acid oxidation
lipid modification
monocarboxylic acid catabolic process
fatty acid catabolic process
fatty acid beta-oxidation
organic acid catabolic process
carboxylic acid catabolic process
small molecule catabolic process
cellular lipid catabolic process
lipid catabolic process
negative regulation of gliogenesis
mitochondrion organization
sphingolipid metabolic process
membrane lipid metabolic process
number of genes
p.adjust

Fig S3: Overlap of differentially expressed parathyroid genes in CKD groups compared to control

(A) Principal component analysis showing separation between samples. (B) Comparing *PNX1* to *ctrl1* 14% of all expressed genes were downregulated and 12% upregulated, which was the case for 10% and 8%, respectively, comparing *PNX2* to *ctrl2*. (C) Venn diagram showing overlapping downregulated genes in CKD groups compared to time-matched controls in the parathyroid glands. Overlapping genes were significantly enriched in terms of mRNA processing by gene ontology analysis, whereas there was no significant enrichment in downregulated genes unique to either CKD group. (D) Venn diagram showing overlapping upregulated genes in CKD groups compared to time-matched controls in the parathyroid glands. Overlapping genes were significantly enriched in terms of ATP synthesis by gene ontology analysis, and genes only upregulated in *PNX2* were enriched in terms of fatty acid metabolism, whereas there was no significant enrichment of genes only upregulated in *PNX1*.

A: GSEA leading-edge *PNX1* vs *ctrl1*

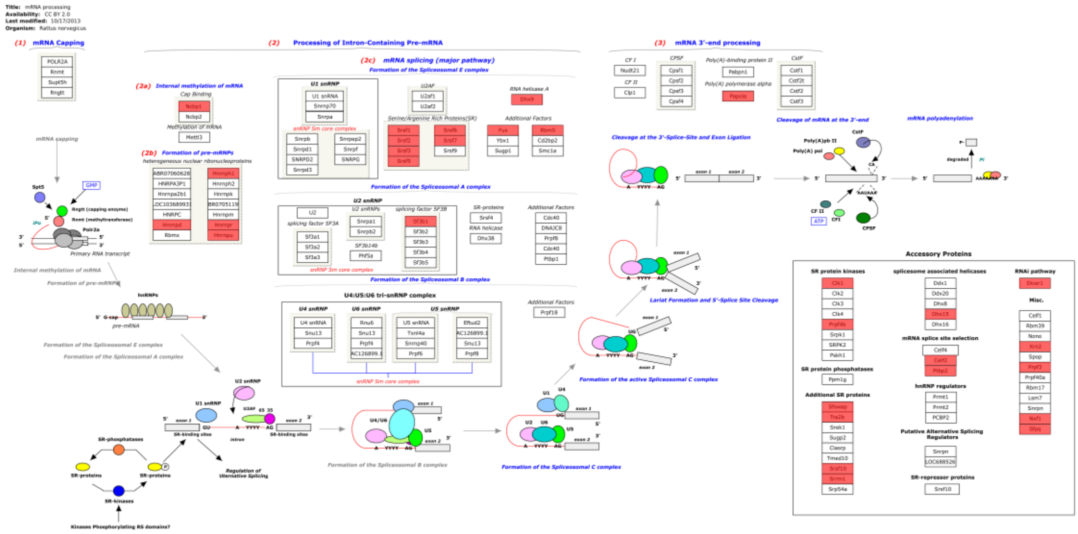

B: GSEA leading-edge *PNX2* vs *ctrl2*

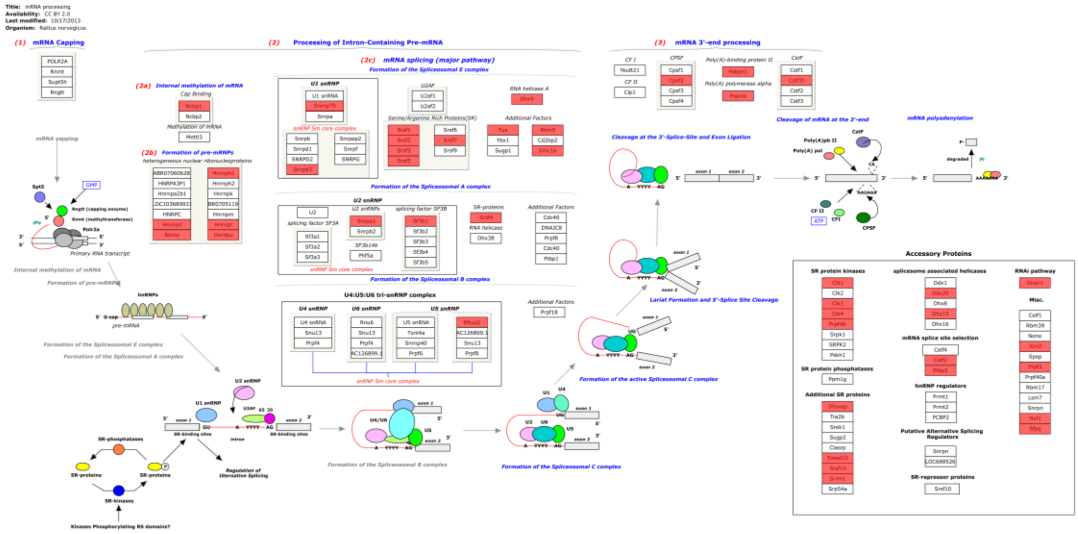

C: GSEA leading-edge *Cina1* vs *PNX1*

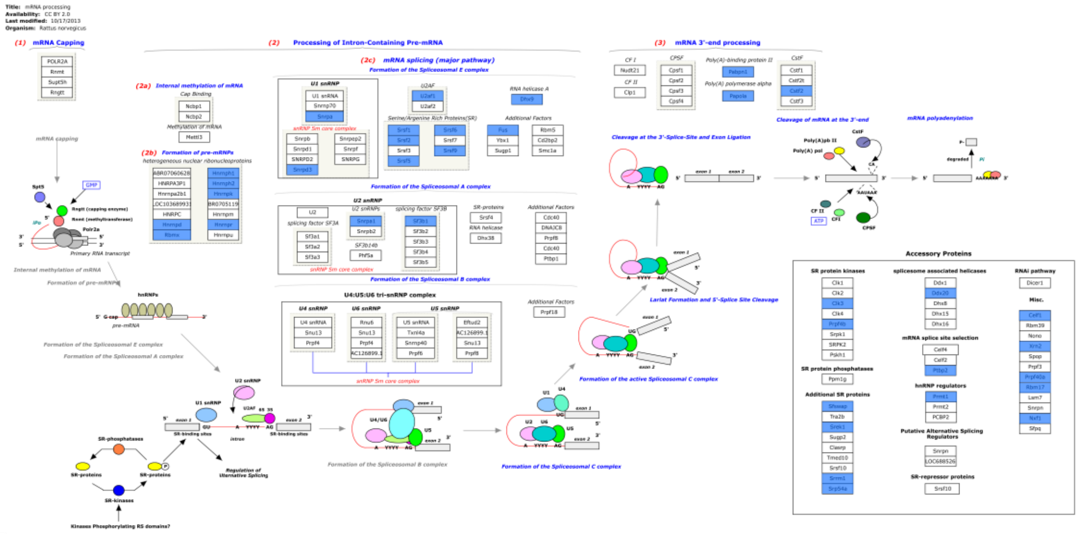

54

Fig S4: Leading-edge genes from GSEA analyses of the pathway: *mRNA processing*

For each of the three GSEA analyses: (A) *PNX1* vs. *ctrl1*, (B) *PNX2* vs. *ctrl2*, and (C) *Cina1* vs. *PNX1,* the resulting leading-edge genes are highlighted onto the WikiPathway WP529 mRNA processing (*Rattus norvegicus*). Blue color denotes upregulated direction. Red color denotes downregulated direction.

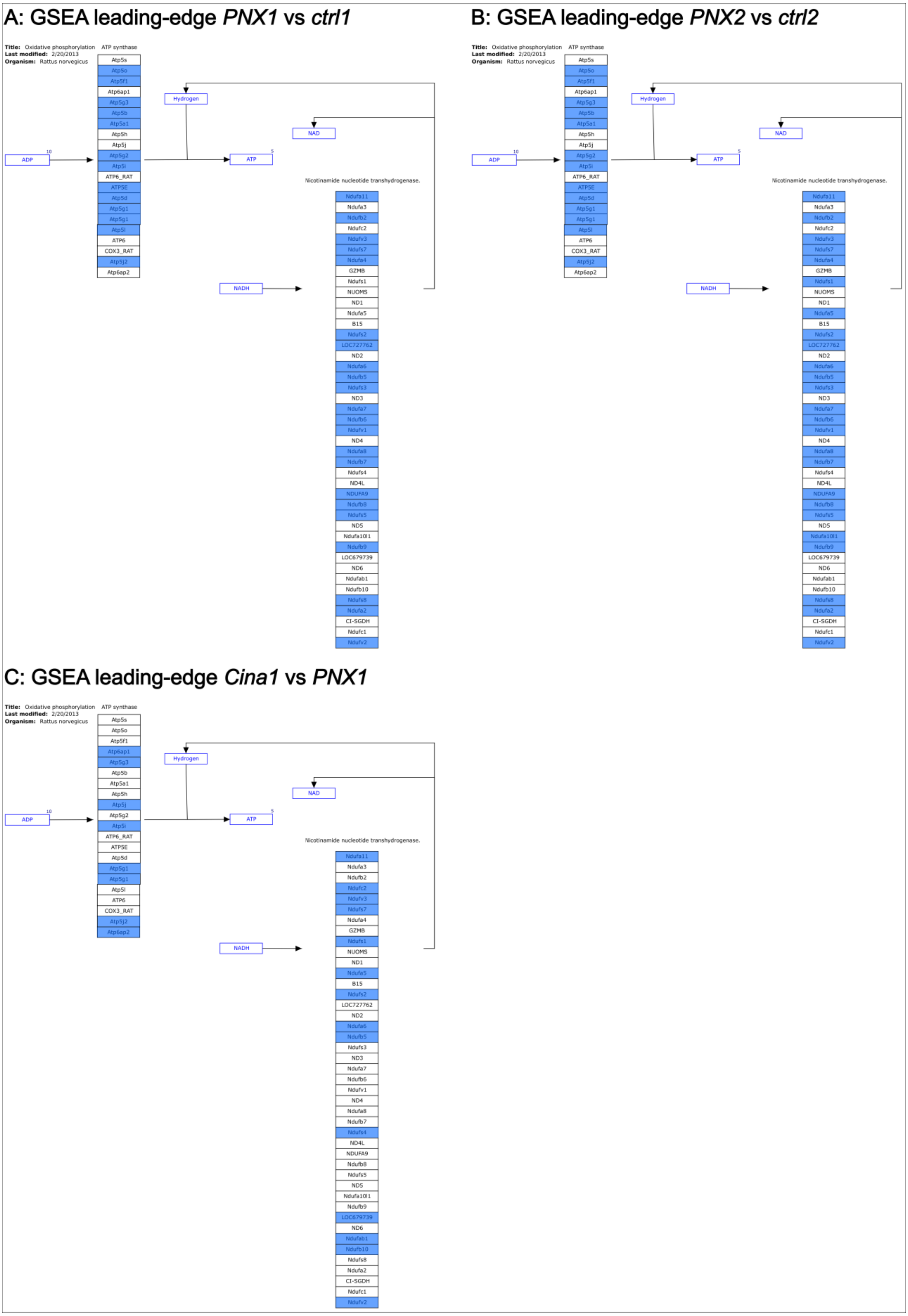
A: GSEA leading-edge PNX1 vs ctrl1
B: GSEA leading-edge PNX2 vs ctrl2
C: GSEA leading-edge Cina1 vs PNX1
Title: Oxidative phosphorylation
Last modified: 2/20/2013
Organism: Rattus norvegicus
ATP synthase
Hydrogen
NAD
ADP
ATP
NADH
Nicotinamide nucleotide transhydrogenase.

61

Fig S5: Leading-edge genes from GSEA analyses of the pathway: *Oxidative phosphorylation*

For each of the three GSEA analyses: (A) *PNX1* vs. *ctrl1*, (B) *PNX2* vs. *ctrl2*, and (C) *Cina1* vs. *PNX1,* the resulting leading-edge genes are highlighted onto the WikiPathway WP1283: Oxidative phosphorylation (*Rattus norvegicus*). Blue color denotes upregulated direction.

SUPPLEMENTARY TABLES

Table S1. Microstructural properties of the left distal femoral metaphysis and mid-diaphysis.

	Morning (ZT2)			Evening (ZT14)		
	ctrl1	*PNX1*	*Cina1*	*ctrl2*	*PNX2*	*Cina2*
Metaphysis						
BV/TV (%)	20 ± 4	35 ± 18	30 ± 16	19 ± 4	38 ± 23	34 ± 13
Tb.Th (mm)	0.079 ± 0.004	0.089 ± 0.012	0.084 ± 0.009	0.080 ± 0.006	0.088 ± 0.011	0.086 ± 0.006
Tb.N (mm^{-1})	2.9 ± 0.5	4.9 ± 2.2	4.5 ± 2.0	2.6 ± 0.5	5.5 ± 2.7	4.9 ± 1.8
Tb.Sp (mm)	0.36 ± 0.07	0.23 ± 0.15	0.26 ± 0.16	0.41 ± 0.08	0.20 ± 0.14[b]	0.24 ± 0.19
SMI	1.53 ± 0.29	0.42 ± 1.51	0.93 ± 1.40	1.55 ± 0.32	-0.04 ± 2.31	0.50 ± 1.05
vBMD (mg/cm^3)	252 ± 41	408 ± 179	351 ± 159	239 ± 45	424 ± 219	398 ± 132
TMD (mg/cm^3)	892 ± 11	918 ± 21[b]	904 ± 21	889 ± 11	914 ± 39	907 ± 13
Diaphysis						
Ct.Po (%)	1.1 ± 0.7	5.7 ± 2.0[a,b]	4.6 ± 2.7[b]	0.5 ± 0.2	9.4 ± 7.8[a,b]	5.4 ± 3.7[a,b]
Ct.Ar (mm^2)	7.9 ± 0.7	7.4 ± 0.6	7.7 ± 0.6	7.8 ± 0.5	7.9 ± 0.4	7.8 ± 0.7
Ma.Ar (mm^2)	4.6 ± 0.6	4.8 ± 0.8	4.7 ± 0.4	4.6 ± 0.6	5.0 ± 0.5	5.3 ± 0.6
Ct.Th (mm)	0.25 ± 0.02	0.19 ± 0.01[a,b]	0.20 ± 0.02[b]	0.26 ± 0.02	0.18 ± 0.03[a,b]	0.20 ± 0.02[a,b]
TMD (mg/cm^3)	1259 ± 10	1259 ± 14	1265 ± 11	1262 ± 12	1243 ± 23	1257 ± 24

BV/TV: bone volume/tissue volume, Tb.Th: trabecular thickness, Tb.N: trabecular number, Tb.Sp: trabecular spacing, SMI: structure model index, vBMD: volumetric bone mineral density, TMD: tissue mineral density, Ct.Po: Cortical porosity, Ct.Ar: Cortical bone area, Ma.Ar: medullary area, Ct.Th: cortical thickness. Data shown as mean ± SD. [a] denotes $p < 0.05$ compared to *ctrl1*. [b] denotes $p < 0.05$ compared to *ctrl2.*

Table S2. Results from Gene Set Enrichment Analysis (GSEA) and Gene Ontology Analysis comparing transcriptomes of the indicated groups.

<u>PNX1 vs ctrl1</u>

GSEA

ID	Description	ES	NES	pvalue	p.adjust
WP529	mRNA processing	-0.6190723	-2.213340	2.754048e-09	2.643886e-07
WP1283	Oxidative phosphorylation	0.7668832	2.179237	5.732702e-09	2.751697e-07

Gene Ontology Analysis - downregulated

ID	Description	GeneRatio	BgRatio	pvalue	p.adjust
GO:0007416	synapse assembly	43/1588	143/12748	1.687916e-08	9.351054e-05
GO:0050684	regulation of mRNA processing	39/1588	131/12748	1.041906e-07	2.429789e-04
GO:1903311	regulation of mRNA metabolic process	62/1588	256/12748	1.315770e-07	2.429789e-04
GO:0051963	regulation of synapse assembly	30/1588	90/12748	2.026152e-07	2.806220e-04
GO:0006397	mRNA processing	81/1588	385/12748	1.054780e-06	1.168696e-03
GO:0043484	regulation of RNA splicing	38/1588	140/12748	1.955604e-06	1.642630e-03
GO:0050808	synapse organization	84/1588	410/12748	2.075526e-06	1.642630e-03
GO:0006376	mRNA splice site selection	13/1588	26/12748	3.570184e-06	2.472352e-03
GO:0016570	histone modification	85/1588	427/12748	5.967333e-06	3.673225e-03
GO:0006402	mRNA catabolic process	48/1588	204/12748	7.864999e-06	4.357210e-03
GO:0000245	spliceosomal complex assembly	17/1588	44/12748	9.560622e-06	4.815077e-03
GO:0019827	stem cell population maintenance	36/1588	140/12748	1.359787e-05	6.277684e-03
GO:0043487	regulation of RNA stability	37/1588	148/12748	2.051440e-05	8.742290e-03
GO:0098727	maintenance of cell number	36/1588	144/12748	2.639843e-05	9.475184e-03
GO:0099003	vesicle-mediated transport in synapse	49/1588	219/12748	2.676999e-05	9.475184e-03
GO:1901888	regulation of cell junction assembly	43/1588	184/12748	2.736515e-05	9.475184e-03
GO:0048024	regulation of mRNA splicing, via spliceosome	27/1588	97/12748	3.551008e-05	1.157211e-02
GO:0006401	RNA catabolic process	52/1588	240/12748	3.878169e-05	1.193614e-02
GO:0018205	peptidyl-lysine modification	68/1588	340/12748	4.300629e-05	1.211433e-02
GO:0043488	regulation of mRNA stability	34/1588	136/12748	4.373405e-05	1.211433e-02

GO:0010586	miRNA metabolic process	11/1588	24/12748	5.717953e-05	1.508450e-02
GO:0043489	RNA stabilization	18/1588	55/12748	7.180760e-05	1.808246e-02
GO:1902414	protein localization to cell junction	27/1588	102/12748	9.205612e-05	2.197319e-02
GO:0007517	muscle organ development	56/1588	273/12748	9.829550e-05	2.197319e-02
GO:0055001	muscle cell development	40/1588	176/12748	9.915698e-05	2.197319e-02
GO:0016358	dendrite development	57/1588	280/12748	1.062042e-04	2.209208e-02
GO:0006475	internal protein amino acid acetylation	35/1588	148/12748	1.149203e-04	2.209208e-02
GO:0018393	internal peptidyl-lysine acetylation	35/1588	148/12748	1.149203e-04	2.209208e-02
GO:0099504	synaptic vesicle cycle	43/1588	195/12748	1.156445e-04	2.209208e-02
GO:0034329	cell junction assembly	69/1588	358/12748	1.249133e-04	2.288373e-02
GO:0050803	regulation of synapse structure or activity	47/1588	220/12748	1.280498e-04	2.288373e-02
GO:0016573	histone acetylation	34/1588	144/12748	1.477405e-04	2.480250e-02
GO:0061013	regulation of mRNA catabolic process	34/1588	144/12748	1.477405e-04	2.480250e-02
GO:0050807	regulation of synapse organization	46/1588	216/12748	1.624029e-04	2.587996e-02
GO:0043414	macromolecule methylation	55/1588	272/12748	1.672613e-04	2.587996e-02
GO:0070252	actin-mediated cell contraction	23/1588	84/12748	1.723655e-04	2.587996e-02
GO:0006479	protein methylation	38/1588	169/12748	1.849993e-04	2.587996e-02
GO:0008213	protein alkylation	38/1588	169/12748	1.849993e-04	2.587996e-02
GO:0009791	post-embryonic development	27/1588	106/12748	1.852635e-04	2.587996e-02
GO:0099173	postsynapse organization	41/1588	187/12748	1.879764e-04	2.587996e-02
GO:0030900	forebrain development	71/1588	376/12748	1.915304e-04	2.587996e-02
GO:0031440	regulation of mRNA 3'-end processing	11/1588	27/12748	2.091187e-04	2.758375e-02
GO:0034968	histone lysine methylation	27/1588	107/12748	2.188902e-04	2.820120e-02
GO:1903313	positive regulation of mRNA metabolic process	28/1588	113/12748	2.347499e-04	2.955715e-02
GO:0018394	peptidyl-lysine acetylation	36/1588	160/12748	2.662947e-04	3.278384e-02
GO:0036465	synaptic vesicle recycling	21/1588	76/12748	2.837124e-04	3.416884e-02
GO:0035418	protein localization to synapse	20/1588	71/12748	2.970924e-04	3.478346e-02
GO:0008380	RNA splicing	64/1588	336/12748	3.041697e-04	3.478346e-02
GO:1902369	negative regulation of RNA catabolic process	19/1588	66/12748	3.076516e-04	3.478346e-02
GO:0006353	DNA-templated transcription, termination	9/1588	20/12748	3.265836e-04	3.568395e-02

Gene Ontology Analysis - upregulated

ID	Description	GeneRatio	BgRatio	pvalue	p.adjust
GO:0009060	aerobic respiration	66/2044	142/12748	1.126210e-17	6.489222e-14
GO:0046034	ATP metabolic process	88/2044	230/12748	1.893119e-16	5.437419e-13
GO:0006091	generation of precursor metabolites and energy	124/2044	379/12748	2.831006e-16	5.437419e-13
GO:0006119	oxidative phosphorylation	51/2044	101/12748	7.900581e-16	1.138079e-12
GO:0045333	cellular respiration	73/2044	184/12748	7.909872e-15	9.115336e-12
GO:0042773	ATP synthesis coupled electron transport	34/2044	58/12748	1.749134e-13	1.439787e-10
GO:0042775	mitochondrial ATP synthesis coupled electron transport	34/2044	58/12748	1.749134e-13	1.439787e-10
GO:0022900	electron transport chain	46/2044	100/12748	1.559746e-12	1.123407e-09
GO:1902600	proton transmembrane transport	47/2044	105/12748	3.041963e-12	1.947532e-09
GO:0019646	aerobic electron transport chain	30/2044	51/12748	4.137303e-12	2.383914e-09
GO:0015980	energy derivation by oxidation of organic compounds	86/2044	263/12748	1.179460e-11	6.178224e-09
GO:0022904	respiratory electron transport chain	37/2044	78/12748	7.755647e-11	3.724003e-08
GO:0007059	chromosome segregation	91/2044	294/12748	8.653151e-11	3.835343e-08
GO:0000070	mitotic sister chromatid segregation	57/2044	153/12748	1.343439e-10	5.529213e-08
GO:0000819	sister chromatid segregation	62/2044	179/12748	6.750180e-10	2.592969e-07
GO:0098813	nuclear chromosome segregation	74/2044	240/12748	6.043095e-09	2.176270e-06
GO:0140014	mitotic nuclear division	78/2044	263/12748	1.613328e-08	5.468232e-06
GO:0051301	cell division	114/2044	442/12748	6.146862e-08	1.967679e-05
GO:0006753	nucleoside phosphate metabolic process	104/2044	397/12748	1.022781e-07	3.101719e-05
GO:0006120	mitochondrial electron transport, NADH to ubiquinone	15/2044	23/12748	1.536333e-07	4.426176e-05
GO:0009150	purine ribonucleotide metabolic process	84/2044	306/12748	2.077831e-07	5.641606e-05
GO:0019693	ribose phosphate metabolic process	89/2044	330/12748	2.154032e-07	5.641606e-05
GO:0000280	nuclear division	100/2044	384/12748	2.447886e-07	5.906724e-05
GO:0055086	nucleobase-containing small molecule metabolic process	113/2044	448/12748	2.460281e-07	5.906724e-05
GO:0009117	nucleotide metabolic process	101/2044	390/12748	2.840544e-07	6.546887e-05
GO:0051983	regulation of chromosome segregation	32/2044	81/12748	3.147767e-07	6.975936e-05
GO:0033047	regulation of mitotic sister chromatid segregation	21/2044	43/12748	5.296913e-07	1.130400e-04
GO:0033046	negative regulation of sister chromatid segregation	20/2044	40/12748	6.098081e-07	1.171238e-04

GO:0033048	negative regulation of mitotic sister chromatid segregation	20/2044	40/12748	6.098081e-07	1.171238e-04
GO:2000816	negative regulation of mitotic sister chromatid separation	20/2044	40/12748	6.098081e-07	1.171238e-04
GO:0007094	mitotic spindle assembly checkpoint signaling	19/2044	37/12748	6.861671e-07	1.198089e-04
GO:0071173	spindle assembly checkpoint signaling	19/2044	37/12748	6.861671e-07	1.198089e-04
GO:0071174	mitotic spindle checkpoint signaling	19/2044	37/12748	6.861671e-07	1.198089e-04
GO:0006163	purine nucleotide metabolic process	86/2044	325/12748	8.265584e-07	1.400773e-04
GO:0046395	carboxylic acid catabolic process	57/2044	190/12748	8.970824e-07	1.439422e-04
GO:0072521	purine-containing compound metabolic process	90/2044	345/12748	8.993263e-07	1.439422e-04
GO:0009259	ribonucleotide metabolic process	85/2044	321/12748	9.275209e-07	1.444426e-04
GO:0015985	energy coupled proton transport, down electrochemical gradient	13/2044	20/12748	1.135200e-06	1.677185e-04
GO:0015986	ATP synthesis coupled proton transport	13/2044	20/12748	1.135200e-06	1.677185e-04
GO:0031577	spindle checkpoint signaling	19/2044	38/12748	1.166568e-06	1.680442e-04
GO:0016054	organic acid catabolic process	57/2044	192/12748	1.302813e-06	1.830928e-04
GO:0009152	purine ribonucleotide biosynthetic process	44/2044	135/12748	1.358183e-06	1.863298e-04
GO:1905819	negative regulation of chromosome separation	20/2044	42/12748	1.641380e-06	2.199449e-04
GO:0044282	small molecule catabolic process	76/2044	282/12748	1.689777e-06	2.212839e-04
GO:0045841	negative regulation of mitotic metaphase/anaphase transition	19/2044	39/12748	1.933906e-06	2.476260e-04
GO:0072522	purine-containing compound biosynthetic process	48/2044	155/12748	2.392520e-06	2.996891e-04
GO:0008608	attachment of spindle microtubules to kinetochore	17/2044	33/12748	2.516397e-06	3.076911e-04
GO:0051985	negative regulation of chromosome segregation	20/2044	43/12748	2.608065e-06	3.076911e-04
GO:0048285	organelle fission	106/2044	433/12748	2.616603e-06	3.076911e-04
GO:0006164	purine nucleotide biosynthetic process	46/2044	148/12748	3.450199e-06	3.976009e-04

PNX2 vs ctrl2

GSEA

ID	Description	ES	NES	pvalue	p.adjust
WP1283	Oxidative phosphorylation	0.7847590	2.305667	1.885412e-09	1.753433e-07
WP529	mRNA processing	-0.5594733	-1.982140	1.296382e-05	3.028174e-04

WP347	TCA cycle	0.7480506	1.946170	6.735891e-05	1.288126e-03
WP372	Beta-oxidation meta-pathway	0.7286142	1.918136	2.002154e-04	3.527507e-03
WP2562	Relationship between glutathione and NADPH	0.6028642	1.804520	2.007051e-04	3.535115e-03
WP504	Fatty acid biosynthesis	0.7077195	1.719390	4.102259e-03	3.852335e-02
WP40	Inflammatory response pathway	0.7267674	1.775494	1.915940e-03	2.221320e-02

Gene Ontology Analysis - downregulated

ID	Description	GeneRatio	BgRatio	pvalue	p.adjust
GO:0016570	histone modification	78/1175	418/11755	3.129579e-08	0.0001629259
GO:1903311	regulation of mRNA metabolic process	53/1175	249/11755	7.164745e-08	0.0001864983
GO:0060537	muscle tissue development	66/1175	346/11755	1.584725e-07	0.0002750027
GO:0006397	mRNA processing	70/1175	380/11755	2.707534e-07	0.0003523855
GO:0014706	striated muscle tissue development	61/1175	324/11755	7.462124e-07	0.0007769564
GO:0035914	skeletal muscle cell differentiation	18/1175	51/11755	9.808632e-07	0.0008510623
GO:0120162	positive regulation of cold-induced thermogenesis	22/1175	73/11755	1.410791e-06	0.0010492253
GO:0043488	regulation of mRNA stability	31/1175	131/11755	3.792153e-06	0.0021705853
GO:0061013	regulation of mRNA catabolic process	32/1175	138/11755	4.224315e-06	0.0021705853
GO:1901379	regulation of potassium ion transmembrane transport	19/1175	61/11755	4.248209e-06	0.0021705853
GO:1990845	adaptive thermogenesis	30/1175	126/11755	4.740205e-06	0.0021705853
GO:0061158	3'-UTR-mediated mRNA destabilization	9/1175	16/11755	5.768804e-06	0.0021705853
GO:0099003	vesicle-mediated transport in synapse	41/1175	200/11755	5.789581e-06	0.0021705853
GO:0035162	embryonic hemopoiesis	12/1175	28/11755	6.246980e-06	0.0021705853
GO:0007416	synapse assembly	31/1175	134/11755	6.254087e-06	0.0021705853
GO:0048568	embryonic organ development	65/1175	378/11755	8.161766e-06	0.0025651067
GO:0120161	regulation of cold-induced thermogenesis	28/1175	117/11755	8.783693e-06	0.0025651067
GO:0061157	mRNA destabilization	21/1175	75/11755	8.868982e-06	0.0025651067
GO:0099504	synaptic vesicle cycle	37/1175	178/11755	1.175751e-05	0.0032202607
GO:0106106	cold-induced thermogenesis	28/1175	119/11755	1.237134e-05	0.0032202607
GO:0043266	regulation of potassium ion transport	20/1175	72/11755	1.643503e-05	0.0038961511
GO:0050779	RNA destabilization	21/1175	78/11755	1.721311e-05	0.0038961511
GO:0061014	positive regulation of mRNA catabolic process	21/1175	78/11755	1.721311e-05	0.0038961511

GO:0007517	muscle organ development	46/1175	245/11755	1.827376e-05	0.0039638830
GO:0035418	protein localization to synapse	19/1175	67/11755	1.928821e-05	0.0040165766
GO:0043487	regulation of RNA stability	31/1175	142/11755	2.159838e-05	0.0043170931
GO:0003007	heart morphogenesis	41/1175	211/11755	2.238984e-05	0.0043170931
GO:0050684	regulation of mRNA processing	29/1175	130/11755	2.572987e-05	0.0044189061
GO:0099560	synaptic membrane adhesion	11/1175	27/11755	2.731617e-05	0.0044189061
GO:0019827	stem cell population maintenance	30/1175	137/11755	2.741553e-05	0.0044189061
GO:0016571	histone methylation	28/1175	124/11755	2.788021e-05	0.0044189061
GO:0008380	RNA splicing	57/1175	331/11755	2.798659e-05	0.0044189061
GO:0048871	multicellular organismal homeostasis	68/1175	416/11755	2.830036e-05	0.0044189061
GO:0007519	skeletal muscle tissue development	31/1175	144/11755	2.885955e-05	0.0044189061
GO:1901016	regulation of potassium ion transmembrane transporter activity	14/1175	43/11755	4.459528e-05	0.0066332292
GO:0006402	mRNA catabolic process	38/1175	196/11755	4.646907e-05	0.0067199432
GO:0098727	maintenance of cell number	30/1175	141/11755	4.873129e-05	0.0068566245
GO:0031056	regulation of histone modification	31/1175	148/11755	5.039132e-05	0.0068940759
GO:0060538	skeletal muscle organ development	32/1175	155/11755	5.164598e-05	0.0068940759
GO:0006476	protein deacetylation	23/1175	96/11755	5.346919e-05	0.0069590151
GO:0050808	synapse organization	62/1175	380/11755	6.699802e-05	0.0085071143
GO:0034968	histone lysine methylation	24/1175	104/11755	7.070768e-05	0.0087643850
GO:0001659	temperature homeostasis	30/1175	144/11755	7.353988e-05	0.0088608789
GO:1902414	protein localization to cell junction	23/1175	98/11755	7.535080e-05	0.0088608789
GO:1903313	positive regulation of mRNA metabolic process	25/1175	111/11755	7.705411e-05	0.0088608789
GO:0097120	receptor localization to synapse	16/1175	56/11755	7.829436e-05	0.0088608789
GO:0043268	positive regulation of potassium ion transport	12/1175	35/11755	8.828615e-05	0.0097790995
GO:0007269	neurotransmitter secretion	29/1175	139/11755	9.379108e-05	0.0099648233
GO:0099643	signal release from synapse	29/1175	139/11755	9.379108e-05	0.0099648233
GO:0046831	regulation of RNA export from nucleus	7/1175	13/11755	9.771920e-05	0.0101745234

Gene Ontology Analysis - upregulated

ID	Description	GeneRatio	BgRatio	pvalue	p.adjust

GO:0009060	aerobic respiration	67/1526	139/11755	3.621288e-24	1.962738e-20
GO:0045333	cellular respiration	75/1526	181/11755	7.354018e-22	1.992939e-18
GO:0006091	generation of precursor metabolites and energy	115/1526	362/11755	2.663895e-21	4.812770e-18
GO:0006119	oxidative phosphorylation	51/1526	98/11755	1.369054e-20	1.855068e-17
GO:0046034	ATP metabolic process	81/1526	216/11755	3.088517e-20	3.347953e-17
GO:0015980	energy derivation by oxidation of organic compounds	86/1526	256/11755	7.099718e-18	6.413412e-15
GO:0042773	ATP synthesis coupled electron transport	34/1526	56/11755	6.074661e-17	4.115583e-14
GO:0042775	mitochondrial ATP synthesis coupled electron transport	34/1526	56/11755	6.074661e-17	4.115583e-14
GO:0022904	respiratory electron transport chain	39/1526	76/11755	8.701435e-16	5.240198e-13
GO:0019646	aerobic electron transport chain	30/1526	49/11755	3.020399e-15	1.637056e-12
GO:0022900	electron transport chain	44/1526	98/11755	6.887817e-15	3.393815e-12
GO:1902600	proton transmembrane transport	41/1526	97/11755	6.857268e-13	3.097199e-10
GO:0046395	carboxylic acid catabolic process	56/1526	172/11755	1.805662e-11	7.528224e-09
GO:0016054	organic acid catabolic process	56/1526	174/11755	3.022633e-11	1.141210e-08
GO:0000070	mitotic sister chromatid segregation	51/1526	151/11755	3.158330e-11	1.141210e-08
GO:0044282	small molecule catabolic process	72/1526	262/11755	1.996407e-10	6.762828e-08
GO:0032787	monocarboxylic acid metabolic process	103/1526	432/11755	2.535830e-10	8.084824e-08
GO:0007059	chromosome segregation	76/1526	286/11755	3.476884e-10	1.046928e-07
GO:0140014	mitotic nuclear division	69/1526	257/11755	1.421056e-09	4.053748e-07
GO:0000819	sister chromatid segregation	53/1526	176/11755	1.539826e-09	4.172929e-07
GO:0007005	mitochondrion organization	105/1526	460/11755	2.311504e-09	5.965881e-07
GO:0006120	mitochondrial electron transport, NADH to ubiquinone	15/1526	22/11755	3.276440e-09	8.071958e-07
GO:0006099	tricarboxylic acid cycle	17/1526	28/11755	4.051069e-09	9.471152e-07
GO:0006635	fatty acid beta-oxidation	28/1526	67/11755	4.193868e-09	9.471152e-07
GO:0015985	energy coupled proton transport, down electrochemical gradient	14/1526	20/11755	6.595127e-09	1.374830e-06
GO:0015986	ATP synthesis coupled proton transport	14/1526	20/11755	6.595127e-09	1.374830e-06
GO:0034440	lipid oxidation	35/1526	98/11755	7.482401e-09	1.502023e-06
GO:0010257	NADH dehydrogenase complex assembly	23/1526	51/11755	1.774440e-08	3.316367e-06
GO:0032981	mitochondrial respiratory chain complex I assembly	23/1526	51/11755	1.774440e-08	3.316367e-06
GO:0019395	fatty acid oxidation	33/1526	93/11755	2.402960e-08	4.235309e-06
GO:0033108	mitochondrial respiratory chain complex assembly	30/1526	80/11755	2.422409e-08	4.235309e-06

GO:0009062	fatty acid catabolic process	32/1526	89/11755	2.716765e-08	4.601520e-06
GO:0098813	nuclear chromosome segregation	61/1526	234/11755	4.184080e-08	6.872034e-06
GO:0009206	purine ribonucleoside triphosphate biosynthetic process	24/1526	57/11755	4.501874e-08	7.176517e-06
GO:0009145	purine nucleoside triphosphate biosynthetic process	24/1526	58/11755	6.741523e-08	1.043973e-05
GO:0006753	nucleoside phosphate metabolic process	84/1526	368/11755	9.647901e-08	1.452545e-05
GO:0072329	monocarboxylic acid catabolic process	33/1526	99/11755	1.348169e-07	1.974885e-05
GO:0006754	ATP biosynthetic process	21/1526	48/11755	1.412390e-07	2.014514e-05
GO:0009152	purine ribonucleotide biosynthetic process	39/1526	128/11755	1.563893e-07	2.173410e-05
GO:0009117	nucleotide metabolic process	82/1526	361/11755	1.714007e-07	2.291377e-05
GO:1901293	nucleoside phosphate biosynthetic process	50/1526	184/11755	1.733329e-07	2.291377e-05
GO:0009150	purine ribonucleotide metabolic process	68/1526	283/11755	2.181929e-07	2.815727e-05
GO:0055086	nucleobase-containing small molecule metabolic process	91/1526	418/11755	2.829794e-07	3.566857e-05
GO:0072522	purine-containing compound biosynthetic process	42/1526	146/11755	3.106828e-07	3.827047e-05
GO:0000280	nuclear division	82/1526	367/11755	3.578298e-07	4.309862e-05
GO:0042776	mitochondrial ATP synthesis coupled proton transport	11/1526	16/11755	3.985756e-07	4.589158e-05
GO:0006631	fatty acid metabolic process	69/1526	293/11755	4.000987e-07	4.589158e-05
GO:0030258	lipid modification	47/1526	173/11755	4.064199e-07	4.589158e-05
GO:0009201	ribonucleoside triphosphate biosynthetic process	24/1526	63/11755	4.292442e-07	4.747967e-05
GO:0006164	purine nucleotide biosynthetic process	40/1526	139/11755	5.847666e-07	6.338870e-05

Cina1 vs PNX1

GSEA

ID	Description	ES	NES	pvalue	p.adjust
WP348	G1 to S cell cycle control	-0.6103480	-1.996948	1.273222e-04	2.367431e-03
WP347	TCA cycle	0.6915914	1.955272	5.259981e-04	9.671294e-03
WP1283	Oxidative phosphorylation	0.5618790	1.817539	6.305248e-04	9.666298e-03
WP303	Prostaglandin synthesis and regulation	0.6405608	1.839993	1.125728e-03	1.143855e-02
WP655	p53 pathway	-0.6049422	-1.823430	1.909887e-03	1.935520e-02

WP529	mRNA processing	0.4197850	1.581453	2.730890e-03	2.130282e-02
WP654	ATM signaling pathway	-0.6677259	-1.817877	2.796763e-03	2.209917e-02
WP656	p53 signal pathway	-0.6430116	-1.781815	7.819253e-03	4.657522e-02

Gene Ontology Analysis - downregulated

ID	Description	GeneRatio	BgRatio	pvalue	p.adjust
GO:0007059	chromosome segregation	17/54	294/12826	1.845325e-15	1.681091e-12
GO:0006334	nucleosome assembly	10/54	78/12826	7.356373e-13	2.239819e-10
GO:0098813	nuclear chromosome segregation	14/54	240/12826	7.375913e-13	2.239819e-10
GO:0051301	cell division	17/54	441/12826	1.413493e-12	3.219231e-10
GO:0000280	nuclear division	16/54	384/12826	2.282315e-12	4.105413e-10
GO:0140014	mitotic nuclear division	14/54	264/12826	2.703894e-12	4.105413e-10
GO:0031497	chromatin assembly	10/54	96/12826	6.232458e-12	8.111099e-10
GO:0000819	sister chromatid segregation	12/54	180/12826	8.296304e-12	9.447416e-10
GO:0048285	organelle fission	16/54	433/12826	1.404861e-11	1.411648e-09
GO:0034728	nucleosome organization	10/54	105/12826	1.549558e-11	1.411648e-09
GO:0000070	mitotic sister chromatid segregation	11/54	154/12826	3.205165e-11	2.654459e-09
GO:0006333	chromatin assembly or disassembly	10/54	114/12826	3.553823e-11	2.697944e-09
GO:0006323	DNA packaging	10/54	126/12826	9.687843e-11	6.788942e-09
GO:0065004	protein-DNA complex assembly	10/54	140/12826	2.760481e-10	1.796284e-08
GO:0071103	DNA conformation change	11/54	209/12826	8.570945e-10	5.205421e-08
GO:0071824	protein-DNA complex subunit organization	10/54	170/12826	1.857391e-09	1.057552e-07
GO:0006338	chromatin remodeling	10/54	176/12826	2.602586e-09	1.394680e-07
GO:0000910	cytokinesis	9/54	154/12826	1.375741e-08	6.962776e-07
GO:0090068	positive regulation of cell cycle process	10/54	214/12826	1.709206e-08	8.195192e-07
GO:0044770	cell cycle phase transition	13/54	444/12826	2.613047e-08	1.190243e-06
GO:0044772	mitotic cell cycle phase transition	12/54	366/12826	2.836474e-08	1.230490e-06
GO:0090307	mitotic spindle assembly	6/54	63/12826	2.365690e-07	9.698699e-06
GO:0006325	chromatin organization	10/54	284/12826	2.448629e-07	9.698699e-06
GO:0045787	positive regulation of cell cycle	10/54	292/12826	3.165945e-07	1.201740e-05

GO:0051225	spindle assembly	7/54	111/12826	3.806076e-07	1.386934e-05
GO:0007052	mitotic spindle organization	7/54	116/12826	5.141808e-07	1.801610e-05
GO:0007346	regulation of mitotic cell cycle	11/54	401/12826	6.943522e-07	2.342796e-05
GO:0007051	spindle organization	8/54	180/12826	7.724296e-07	2.513155e-05
GO:0032465	regulation of cytokinesis	6/54	78/12826	8.521968e-07	2.593988e-05
GO:0007093	mitotic cell cycle checkpoint signaling	7/54	125/12826	8.542222e-07	2.593988e-05
GO:0051983	regulation of chromosome segregation	6/54	82/12826	1.146945e-06	3.370540e-05
GO:1901987	regulation of cell cycle phase transition	10/54	338/12826	1.207432e-06	3.437407e-05
GO:0010948	negative regulation of cell cycle process	9/54	261/12826	1.245509e-06	3.438360e-05
GO:1901990	regulation of mitotic cell cycle phase transition	9/54	263/12826	1.327014e-06	3.555263e-05
GO:0044839	cell cycle G2/M phase transition	7/54	134/12826	1.365908e-06	3.555263e-05
GO:0006261	DNA-dependent DNA replication	7/54	138/12826	1.664509e-06	4.212132e-05
GO:1902850	microtubule cytoskeleton organization involved in mitosis	7/54	141/12826	1.922748e-06	4.734118e-05
GO:0045930	negative regulation of mitotic cell cycle	8/54	206/12826	2.133585e-06	5.114990e-05
GO:1901988	negative regulation of cell cycle phase transition	8/54	220/12826	3.482296e-06	8.134285e-05
GO:1901991	negative regulation of mitotic cell cycle phase transition	7/54	157/12826	3.936404e-06	8.965161e-05
GO:0006260	DNA replication	8/54	228/12826	4.537127e-06	1.008127e-04
GO:0000075	cell cycle checkpoint signaling	7/54	162/12826	4.844586e-06	1.050814e-04
GO:0006310	DNA recombination	8/54	244/12826	7.475770e-06	1.583820e-04
GO:0033045	regulation of sister chromatid segregation	5/54	67/12826	8.672450e-06	1.762179e-04
GO:0045786	negative regulation of cell cycle	9/54	331/12826	8.704507e-06	1.762179e-04
GO:0016584	nucleosome positioning	3/54	11/12826	1.136608e-05	2.203084e-04
GO:0051255	spindle midzone assembly	3/54	11/12826	1.136608e-05	2.203084e-04
GO:0000086	G2/M transition of mitotic cell cycle	6/54	123/12826	1.220041e-05	2.315536e-04
GO:0045132	meiotic chromosome segregation	5/54	76/12826	1.612149e-05	2.997280e-04
GO:0006335	DNA replication-dependent nucleosome assembly	3/54	13/12826	1.958398e-05	3.498236e-04

Gene Ontology Analysis - upregulated

ID	Description	GeneRatio	BgRatio	pvalue	p.adjust

<0 rows>

Cina2 vs PNX2

GSEA

ID	Description	ES	NES	pvalue	p.adjust
WP654	ATM signaling pathway	-0.6977680	-2.093226	2.189909e-04	0.0063407709
WP655	p53 pathway	-0.5858091	-1.950821	7.174001e-04	0.0181176035
WP348	G1 to S cell cycle control	-0.4665046	-1.694029	3.365712e-03	0.0479113876

Gene Ontology Analysis - downregulated

ID	Description	GeneRatio	BgRatio	pvalue	p.adjust

<0 rows>

Gene Ontology Analysis - upregulated

ID	Description	GeneRatio	BgRatio	pvalue	p.adjust

<0 rows>

Table S3. Overview of concordant significant changes identified by RNAseq analysis.

	PNX1 vs. ctrl1	*PNX2 vs. ctrl2*	*Cina1 vs. PNX1*	*Cina2 vs. PNX2*
GO: *mRNA processing*	↓	↓	-	-
GSEA: *mRNA processing*	↓	↓	↑	-
GO: *Oxidative phosphorylation*	↑	↑	-	-
GSEA: *Oxidative phosphorylation*	↑	↑	↑	-
GO: *Mitotic nuclear division*	↑	↑	↓	-
GSEA: *ATM signaling pathway*	-	-	↓	↓
GSEA: *p53 signaling pathway*	-	-	↓	↓
GSEA: *G1 to S cell cycle control*	-	-	↓	↓

Direction of significant (adjusted p-value <0.05) changes identified in ≥ 2 group comparisons by either Gene Ontology analysis or Gene Set Enrichment Analysis of RNAseq data are shown. For details, please refer to the result section.
GO: Gene Ontology analysis, GSEA: Gene Set Enrichment Analysis.
